# Supplementary material for: The non-canonical thioreductase Tmx2b is essential for neuronal survival during zebrafish embryonic brain development
Source: Development. 2025 Sep 18;152(18):dev204348. doi: 10.1242/dev.204348 (PMC12516324; doi:10.1242/dev.204348)
Supplement: Supplementary information [file develop-152-204348-s1.pdf]

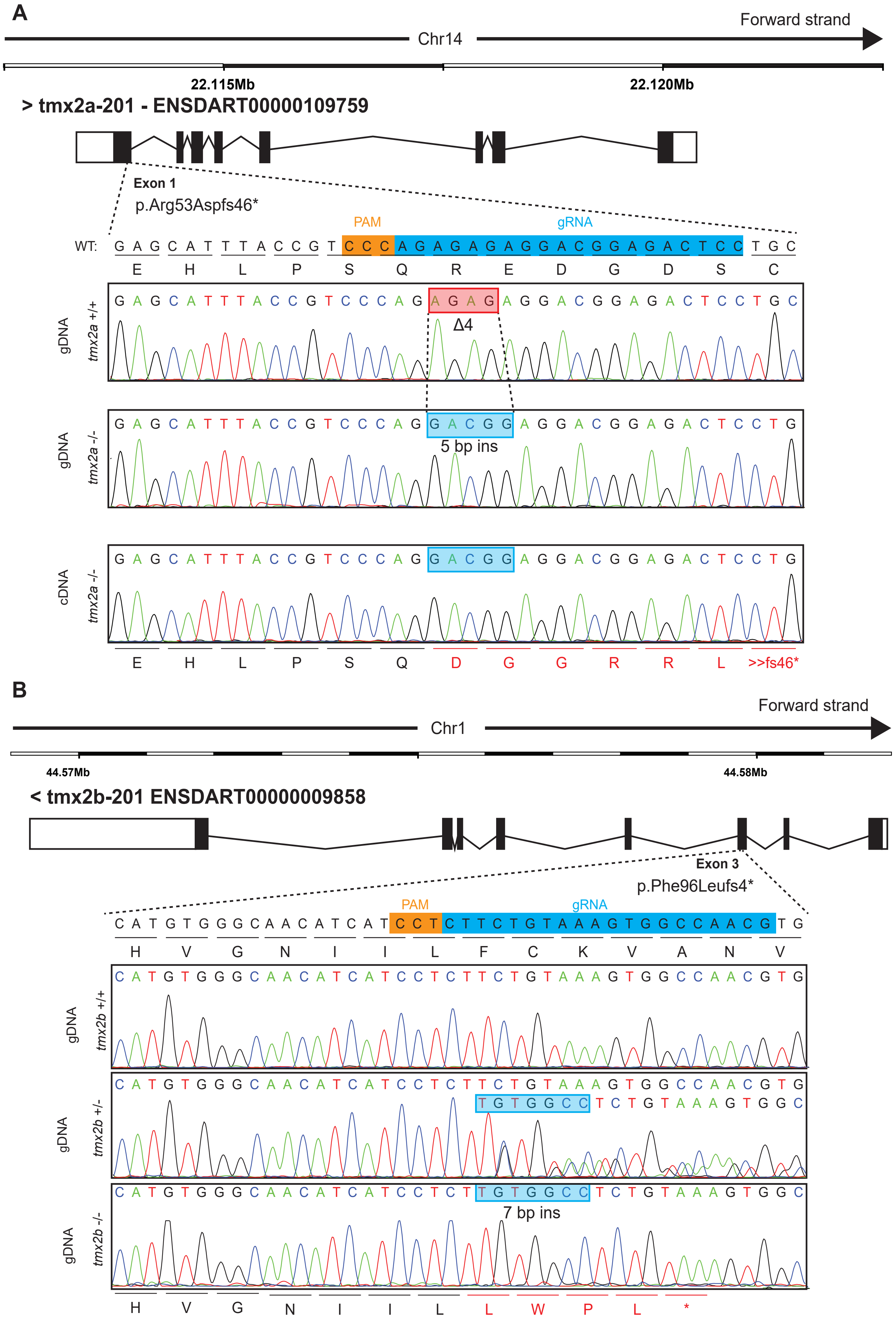

Fig. S1. *tmx2a* and *tmx2b* mutagenesis with CRISPR-Cas9. (A) Schematic representation of the tmx2a gene (Ensembl transcript ID:ENSDART00000109759.5) the gRNA was designed to target exon 1. The adult tmx2a$^{-/-}$ zebrafish had a homozygous c.157_160delinsGACGG, p.Arg53Aspfs46* mutation. (B) Schematic representation of the tmx2b gene (Ensembl transcript ID:ENSDART00000009858.6). the gRNA was designed to target exon 3. The adult tmx2b$^{-/-}$ zebrafish had a heterozygous c.285_286insTGTGGCC, p.Phe96Leufs4* mutation.

Development • Supplementary information

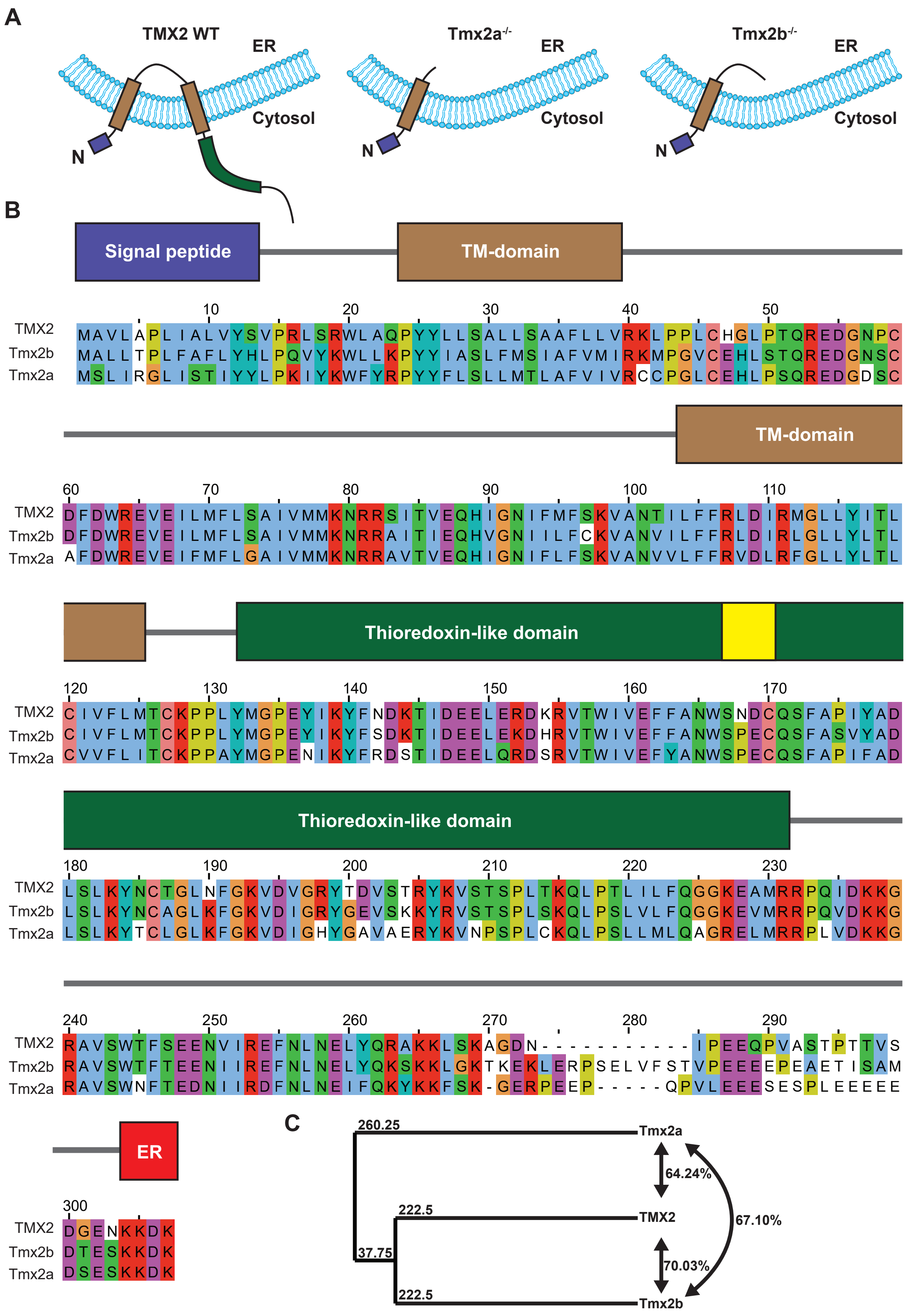

Fig. S2. TMX2, Tmx2a and Tmx2b protein comparison. (A) Schematic representation of TMX2 orientation in the ER membrane. The potential proteins generated from the mutant alleles only contain the N-terminal signal peptide and first transmembrane domain and lack the catalytic domain of the protein. (B) Protein alignment of Tmx2, Tmx2a and Tmx2b by the Jalview v2.11.2.7 software. Color coding is according to the Clustal algorithm. The protein domains drawn above the protein sequence are based on the human TMX2 protein (Interpro ID: Q9Y320). The yellow box indicates the catalytic S-X-X-C site of TMX2 (C) Average distance tree of the BLOSUM62 algorithm, indicating that the Tmx2b is closer related to TMX2 than Tmx2a. Percentages right side indicate protein sequence similarity. Abbreviations: ER, ER retention signal; TM, transmembrane domain.

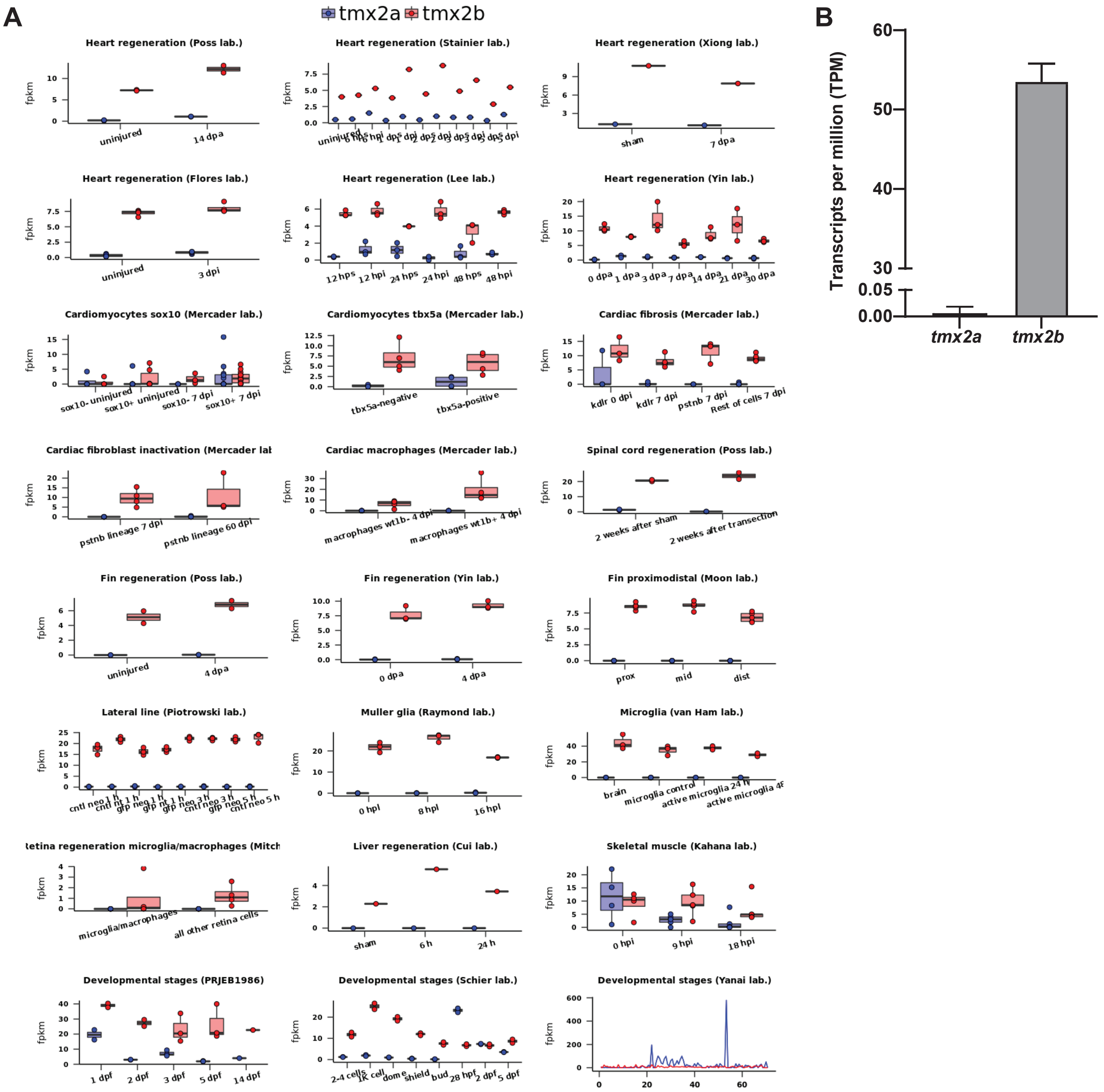

Fig. S3. Tmx2b is the homologue of TMX2. (A) Expression level plots of tmx2a and tmx2b from different RNA-seq data-sets (source: http://zfregeneration.org/). all different data-sets show that tmx2b is the mainly expressed gene. (B) In house RNA-seq data of WT zebrafish brain at 5 days post fertilization (dpf) showing that tmx2a has no expres-sion and tmx2b has an average expression > 50 TPM (n=3, each sample is a pool of brain (sample 1 = 16 brains, sample 2 = 22 brains and sample 3 = 25 brains).

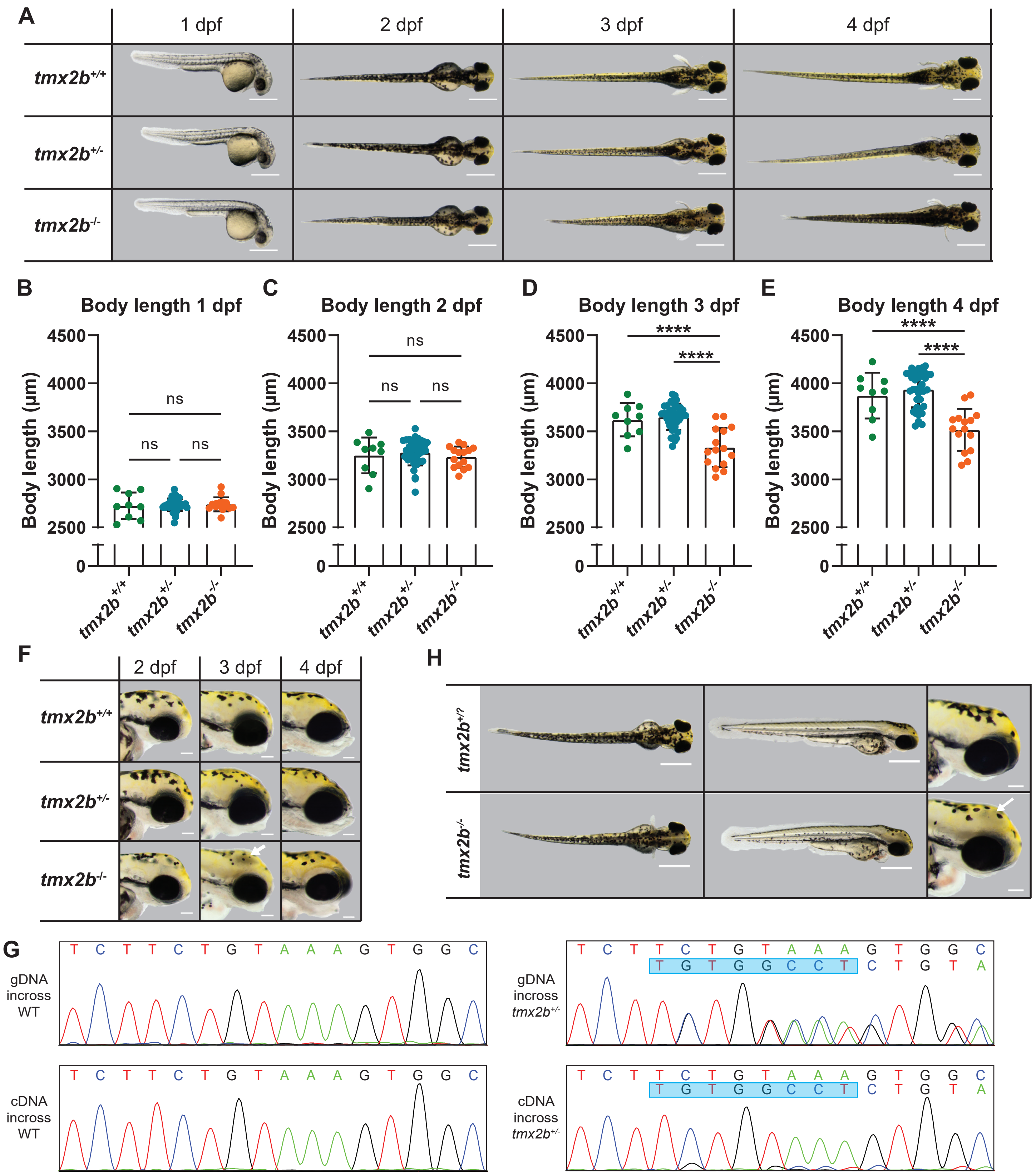

Fig. S4. *tmx2b⁻/⁻* zebrafish display a developmental decline from 3 dpf onwards. (A) Representative images of same tmx2b $^{+/+}$, tmx2b$^{+/-}$ and tmx2b$^{-/-}$ zebrafish from 1 to 4 days post fertilization (dpf). tmx2b$^{-/-}$ have normal body morphology till 2 dpf and from 3 dpf onwards display a developmental decline. Scale bars indicate 500 µm. (B,C,D,E) Body length measurements of tmx2b$^{+/+}$, tmx2b$^{+/-}$ and tmx2b$^{-/-}$ zebrafish from 1 till 4 dpf. tmx2b$^{-/-}$ zebrafish have a small-er body length indicative for a developmental delay from 3 dpf onwards. tmx2b$^{+/+}$ n=9, tmx2b$^{+/-}$ n=37, tmx2b$^{-/-}$ n=15 zebrafish, one-way ANOVA with Tukey's multiple comparisons test. (F) Representative images of tmx2b$^{+/+}$, tmx2b$^{+/-}$ and tmx2b$^{-/-}$ zebrafish brightfield images lateral view of head. tmx2b$^{-/-}$ zebrafish develop a gray discoloration in brain region (white arrow) indicative of necrosis. This gray discoloration is not always present at 4 dpf in tmx2b-/- zebrafish. Scale bars indicate 100 µm (G) Sanger sequencing of genomic and cDNA of pooled zebrafish embryos at 2 dpf from either a WT incross or tmx2b$^{+/-}$ incross. tmx2b$^{+/-}$ incross zebrafish embryos show low peaks of the mutant 7bp inser-tion allele, indicating mRNA decay. (H) Images showing the onset of visible necrosis (white arrow) in the brain of tmx2b$^{-/-}$ zebrafish. Onset of the brain necrosis occurs between the pec-fin stage (60 hpf) and protruding-mouth stage (72 hpf); e.g. embryonic to larval transition. Images in A and F are also shown in Fig. 1C,E. Images in A and F are also shown in Fig. 1C,E. Scale bars whole fish indicate 500 µm. Scale bars fish head indicate 100 µm. Data are represented as mean ± SD. *p < 0.05, **p < 0.01, ***p < 0.001, ****p < 0.0001.

Development • Supplementary information

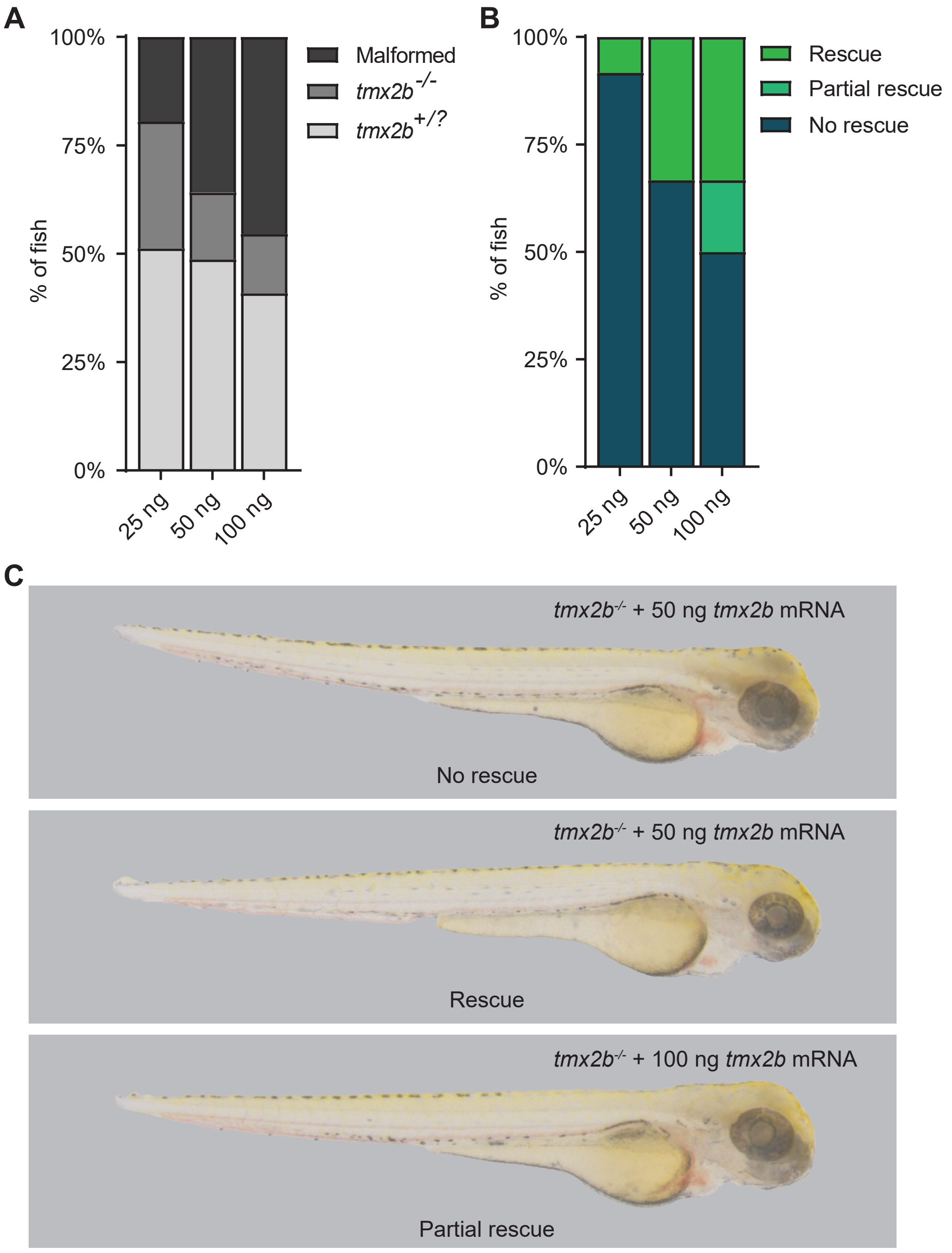

Fig. S5. *tmx2b* mRNA rescues brain necrosis phenotype in *tmx2b*$^{-/-}$ zebrafish (A) Injection of 25 ng, 50 ng or 100ng WT tmx2b mRNA into the one-cell stage of zebrafish embryos is toxic, which is independent of the genotype. (B.C) WT tmx2b mRNA rescues the brain necrosis phenotype in tmx2b$^{-/-}$ zebrafish at 3 dpf. Data in bar graph (B) only shows rescue in tmx2b$^{-/-}$ zebrafish. 25ng, 50ng, 100ng: n=11, 6, 6.

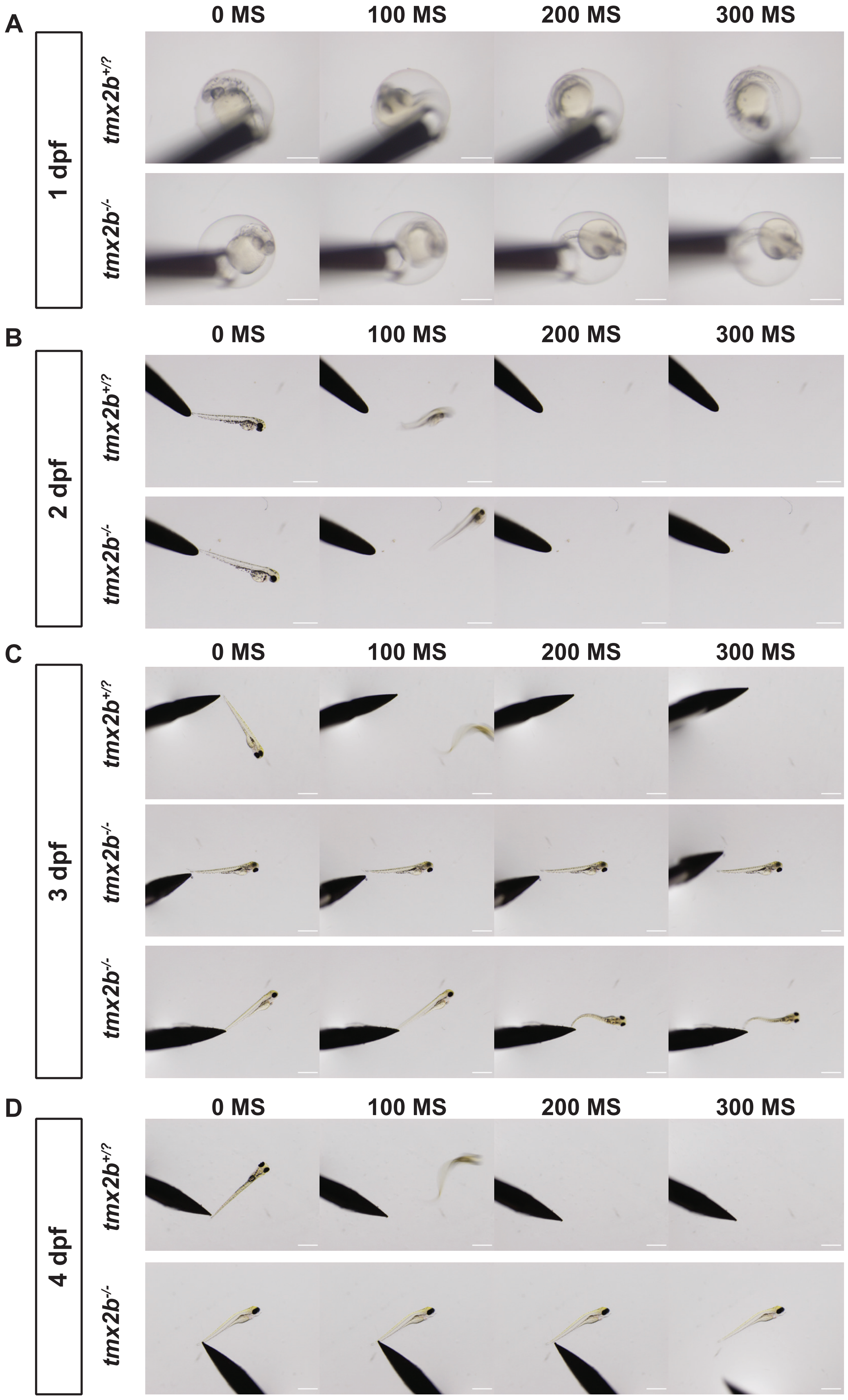

Fig. S6. Touch response assay from 1 till 4 dpf. (A) Touch response at 1 dpf. Touch response was normal if the zebrafish had an immediate twitching movement upon touch. Both tmx2b$^{+/?}$ and tmx2b$^{-/-}$ zebrafish display a normal touch response at 1 dpf. (B) Touch response at 2 dpf. Both tmx2b$^{+/?}$ and tmx2b$^{-/-}$ zebrafish display a normal touch response and immediately swim away upon touch by needle. (C) Touch response at 3 dpf. tmx2b$^{+/?}$ zebrafish immediately swim away upon touch by needle. tmx2b$^{-/-}$ zebrafish no longer swim away upon touch. Middle panel shows a zebrafish with no movements and lower panel a fish with ineffective movements (classified under delayed respose). (D) Touch response at 4 dpf. Similar to 3 dpf, tmx2b$^{+/?}$ zebfrafish swims away upon touch and tmx2b$^{-/-}$ zebrafish are unable to move. tmx2b$^{+/?}$ n=58, tmx2b$^{-/-}$ n=27 zebrafish. Scale bars 1 dpf indicate 500 μm. Scale bars 2-4 dpf indicates 1000 μm.

Development • Supplementary information

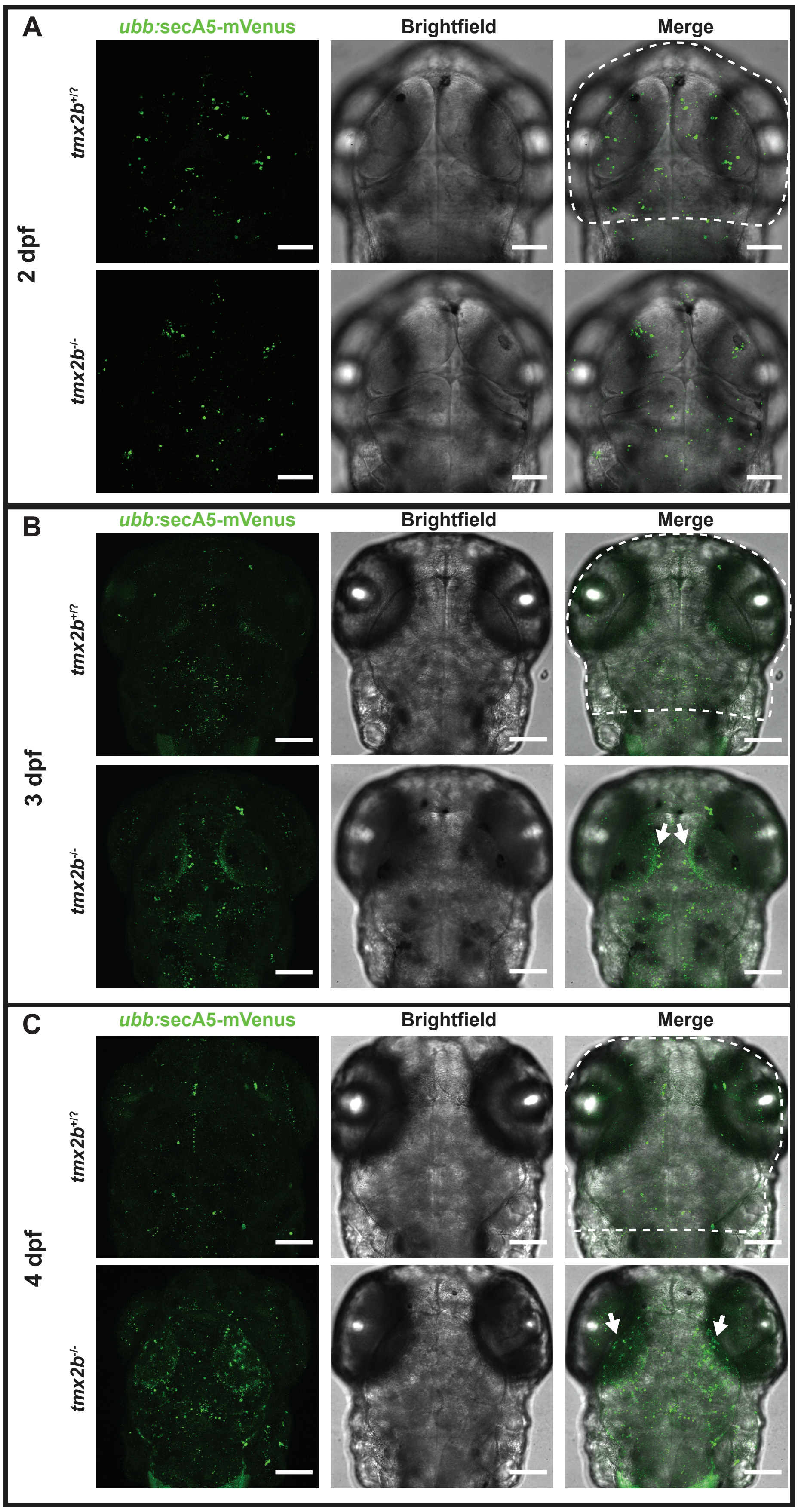

Fig. S7. Apoptotic clusters are increased in *tmx2b$^{-/-}$* brain at 3 and 4 dpf (A,B,C) Representative images of ubb:secA5-mVenus+ (green) apoptotic clusters in tmx2b$^{+/?}$ and tmx2b$^{-/-}$ zebrafish at 2, 3 and 4 dpf. Dashed lines in merged images indicate brain area where the apoptotic clusters were counted. tmx2b$^{-/-}$ zebrafish have an increased number of apoptotic clusters in the brain more pronounced in the optic tecti (white arrows) at 3 and 4 dpf. Images in this figure are also shown in Fig. 1H. Images in this figure are also shown in Fig. 1H. 2 dpf: tmx2b$^{+/?}$ n=30, tmx2b$^{-/-}$ n=6 zebrafish. 3 dpf: tmx2b$^{+/?}$ n=30, tmx2b$^{-/-}$ n=9 zebrafish. 4 dpf: tmx2b$^{+/?}$ n=11, tmx2b$^{-/-}$ n=6 zebrafish. Scale bars 2 dpf indicate 75 µm. Scale bars 3 dpf indicate 100 µm.

Development • Supplementary information

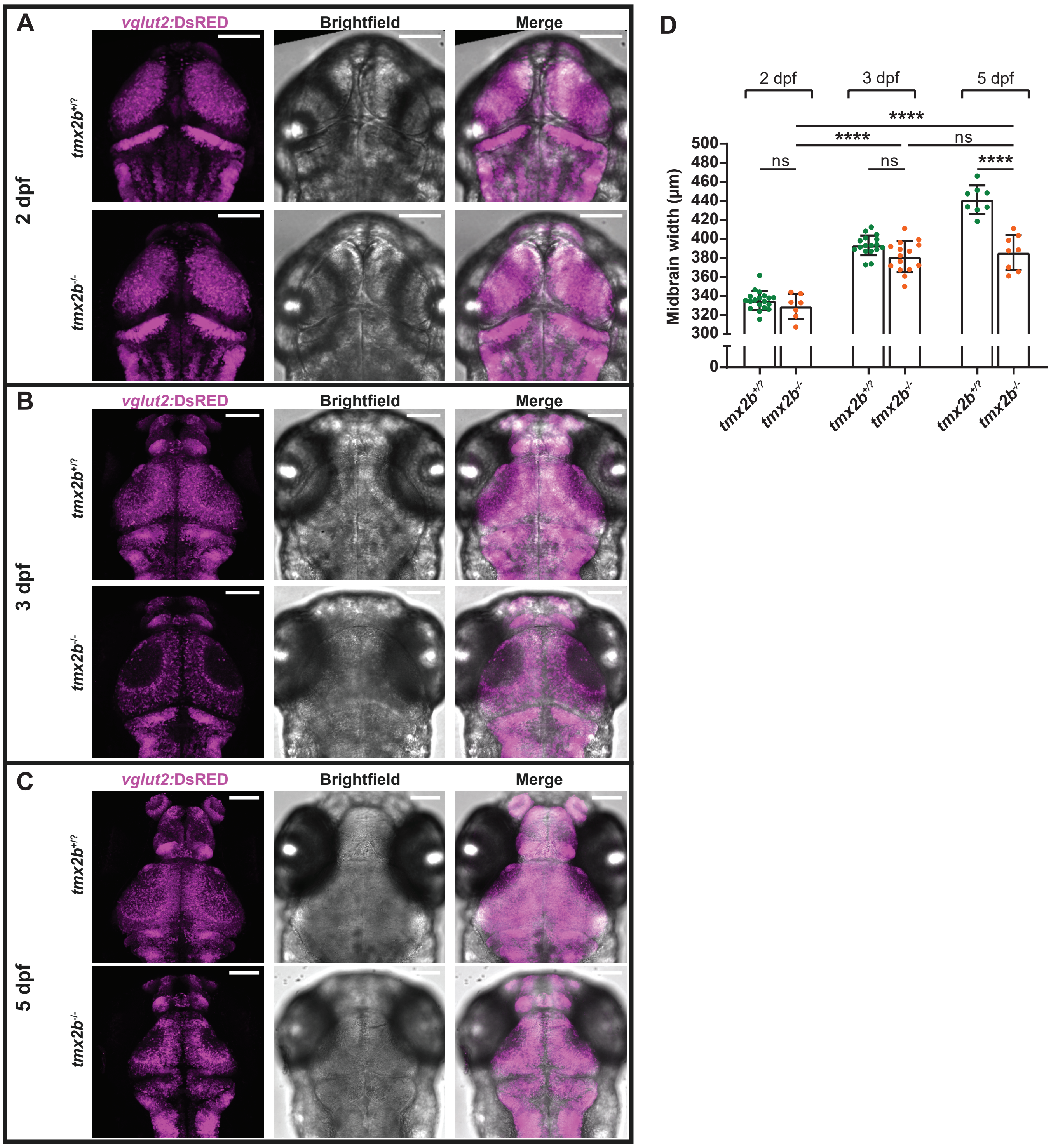

Fig. S8. Excitatory neurons in *$tmx2b^{-/-}$* zebrafish brain undergo cell death between 2 and 3 dpf. (A,B,C) Representative images of vglut2:DsRED+ (magenta) excitatory neurons in $tmx2b^{+/?}$ and $tmx2b^{-/-}$ zebrafish at 2, 3 and 5 dpf. $tmx2b^{-/-}$ zebrafish display excitatory neuronal cell loss at 3 dpf. Scale bars indicate 100 µm. (D) Midbrain width measurements $tmx2b^{+/?}$ and $tmx2b^{-/-}$ zebrafish at 2, 3 and 5 dpf. The largest diameter of the excitatory neurons was measured for the midbrain width. 2 dpf: $tmx2b^{+/?}$ n=19, $tmx2b^{-/-}$ n=7 zebrafish. 3 dpf: $tmx2b^{+/?}$ n=17, $tmx2b^{-/-}$ n=15 zebrafish. 5 dpf: $tmx2b^{+/?}$ n=8, $tmx2b^{-/-}$ n=8 zebrafish. Two-way ANOVA, Tukey's multiple comparisons test. Data are represented as mean ± SD. *p < 0.05, **p < 0.01, ***p < 0.001, ****p < 0.0001.

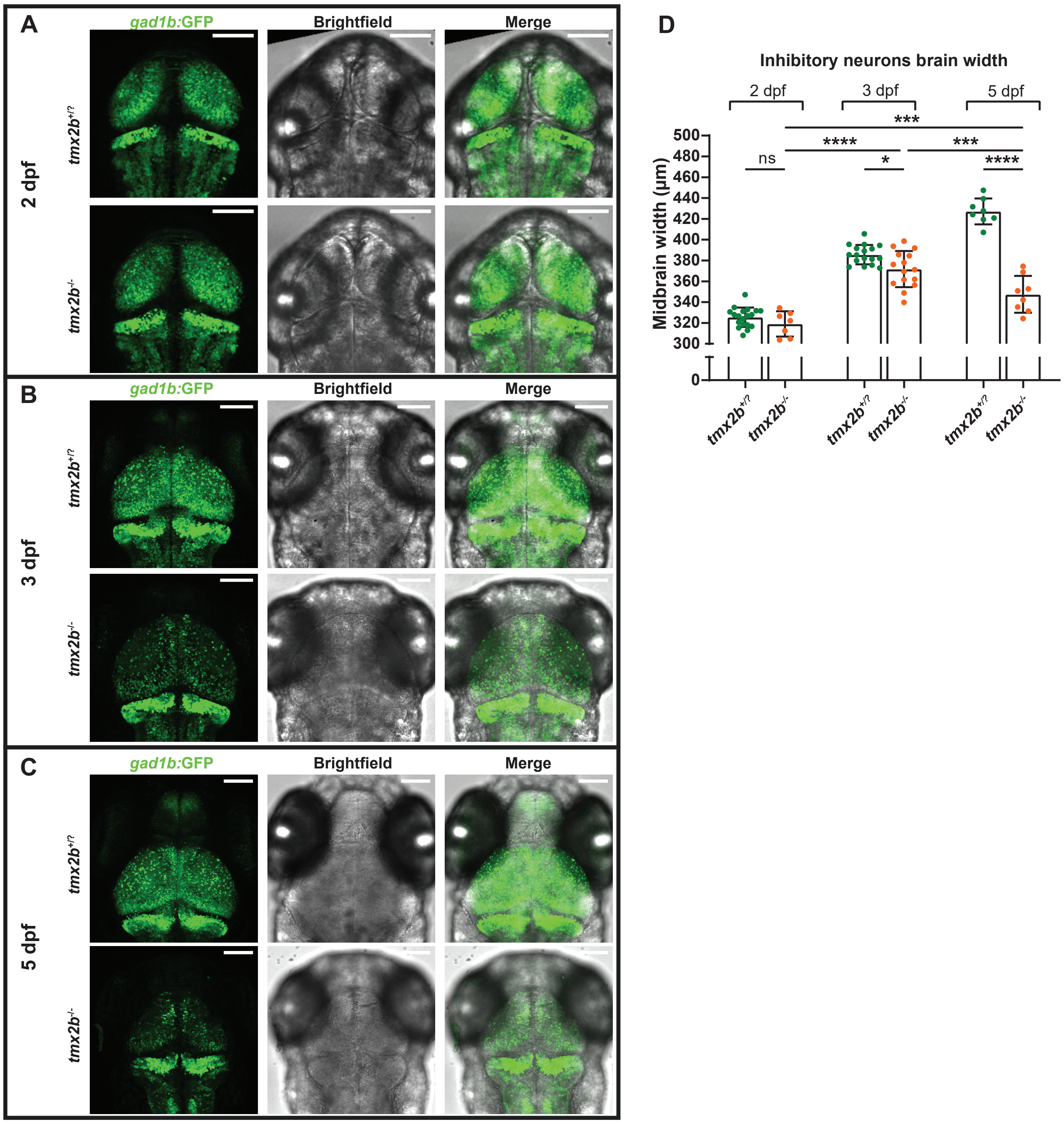

Fig. S9. Inhibitory neurons in in *tmx2b$^{-/-}$* zebrafish brain undergo cell death between 2 and 3 dpf. (A,B,C) Repre-sentative images of gad1b:GFP+ (green) inhibitory neurons in tmx2b$^{+/?}$ and tmx2b$^{-/-}$ zebrafish at 2, 3 and 5 dpf. tmx2b$^{-/-}$ zebrafish display inhibitory neuronal cell loss at 3 dpf. Scale bars indicate 100 µm. (D) Midbrain width measurements tmx2b$^{+/?}$ and tmx2b$^{-/-}$ zebrafish at 2, 3 and 5 dpf. The largest diameter of the inhibitory neurons was measured for the midbrain width. 2 dpf: tmx2b$^{+/?}$ n=19, tmx2b$^{-/-}$ n=7 zebrafish. 3 dpf: tmx2b$^{+/?}$ n=17, tmx2b$^{-/-}$ n=15 zebrafish. 5 dpf: tmx2b$^{+/?}$ n=8, tmx2b$^{-/-}$ n=8 zebrafish. Two-way ANOVA, Tukey's multiple comparisons test. *$p < 0.05$, ***$p < 0.001$, ****$p < 0.0001$.

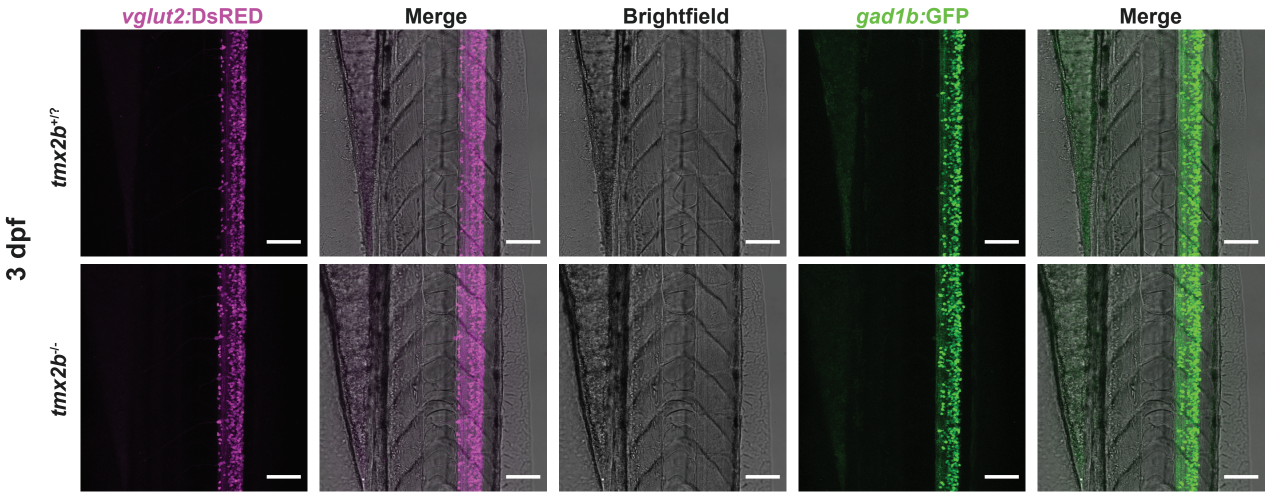

Fig. S10. Excitatory and inhibitory neurons in in *tmx2b*$^{-/-}$ zebrafish spinal cord are unaffected. Representative images of *vglut2:*DsRED+ (magenta) excitatory and *gad1b:*GFP+ (green) inhibitory neurons in spinal cord in t*mx2b*$^{+/?}$ and t*mx2b*$^{-/-}$ zebrafish at 3 dpf. Contrary to the central brain regions the neurons in the spinal cord are unaffected by Tmx2 loss. t*mx2b*$^{+/?}$ n=15, t*mx2b*$^{-/-}$ n=10 zebrafish. Scale bars indicate 75 μm.

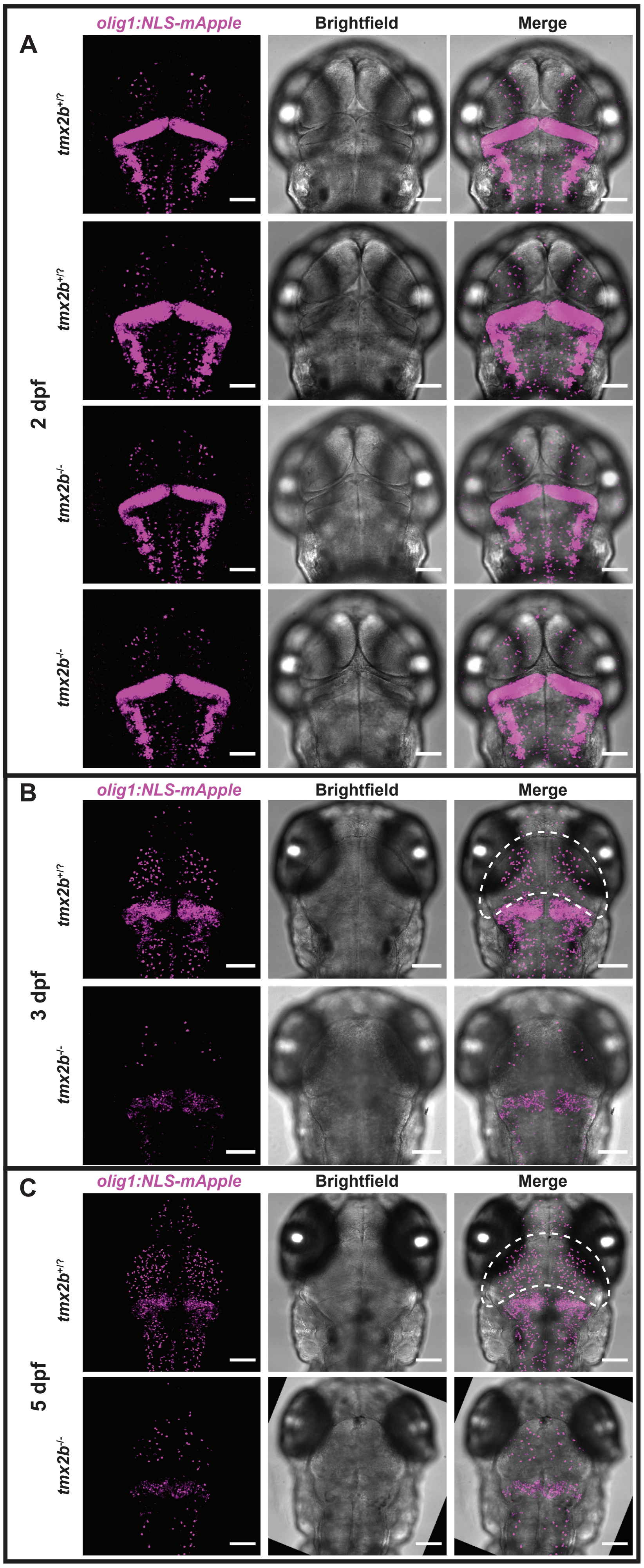

Fig. S11. Oligodendrocyte precursor cells (OPC) are decreased in *tmx2b$^{-/-}$* zebrafish brain. (A,B,C) Representative images of olig1:NLS-mApple (magenta) OPCs in tmx2b$^{+/?}$ and tmx2b$^{-/-}$ zebrafish at 2, 3 and 5 dpf. Scale bars 2 dpf indicate 75 µm. Dashed line indicates measured area. Scale bars at 3 and 5 dpf indicate 100 µm. 2 dpf: tmx2b$^{+/?}$ n=13, tmx2b$^{-/-}$ n=3 zebrafish. 3 dpf: tmx2b$^{+/?}$ n=9, tmx2b$^{-/-}$ n=6 zebrafish. 5 dpf: tmx2b$^{+/?}$ n=7, tmx2b$^{-/-}$ n=3 zebrafish.

Development • Supplementary information

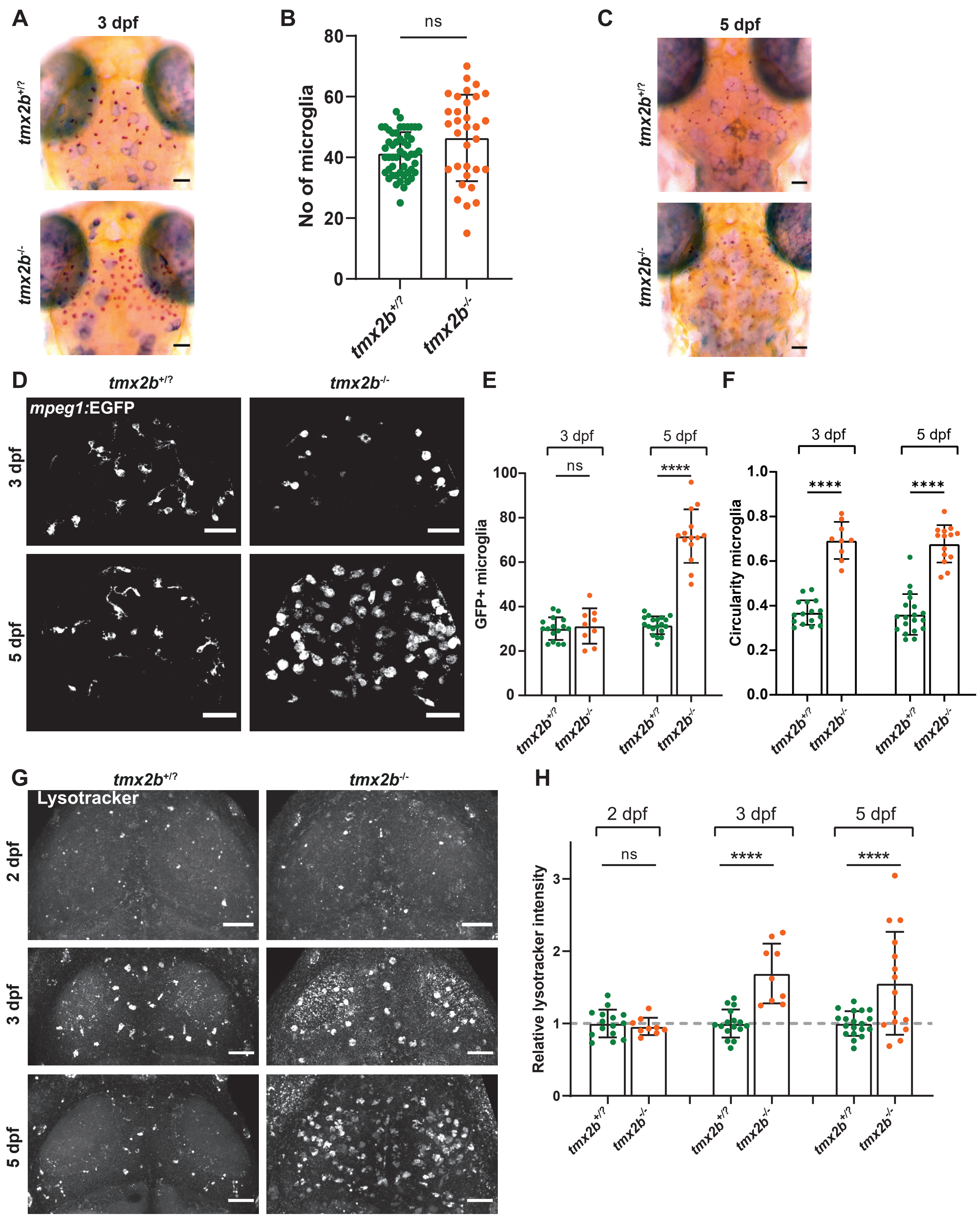

Fig. S12. Microglia activation and increased lysosome numbers in *tmx2b$^{-/-}$* zebrafish (A) Representative images of 3 dpf tmx2b$^{+/?}$ and tmx2b$^{-/-}$ zebrafish after neutral red (NR) staining. Scale bars indicate 100 µm. (B) Quantification of NR+ microglia at 3dpf. tmx2b$^{+/?}$ n=51 tmx2b$^{-/-}$ n=32. Unpaired t-test with Welch's correction. (C) Representative images of 5 dpf tmx2b$^{+/?}$ and tmx2b$^{-/-}$ zebrafish after neutral red (NR) staining. Scale bars indicate 100 µm. (D) Representative images of mpeg1:EGFP+ microglia in midbrain of tmx2b$^{+/?}$ and tmx2b$^{-/-}$ zebrafish at 3 and 5 dpf. Scale bars indicate 50 µm. (E) Quantification of mpeg1:EGFP+ microglia number in midbrain of tmx2b$^{+/?}$ and tmx2b$^{-/-}$ zebrafish at 3 and 5 dpf. (F) Circularity measurement of microglia in midbrain of tmx2b$^{+/?}$ and tmx2b$^{-/-}$ zebrafish at 3 and 5 dpf. Each dot represents the average value of six microglia from a single zebrafish brain. (E,F) 3,5 dpf: tmx2b$^{+/?}$ n=16,19, tmx2b$^{-/-}$ n=9,13 zebrafish. Two-way ANOVA, Šídák's multiple comparisons test. (G) Representative images of lysotracker+ lysosomes in midbrain of tmx2b$^{+/?}$ and tmx2b$^{-/-}$ zebrafish at 2,3 and 5 dpf. Scale bars indicate 50 µm. (H) Relative lysotracker fluoresence intensity normalized to the average tmx2b$^{+/?}$ at 2,3 or 5 dpf. 2,3,5 dpf: tmx2b$^{+/?}$ n=15, 16, 19, tmx2b$^{-/-}$ n=9, 9, 15 zebrafish. Two-way ANOVA, Šídák's multiple comparisons test. Data are represented as mean ± SD. *p < 0.05, **p < 0.01, ***p < 0.001, ****p < 0.0001.

Development • Supplementary information

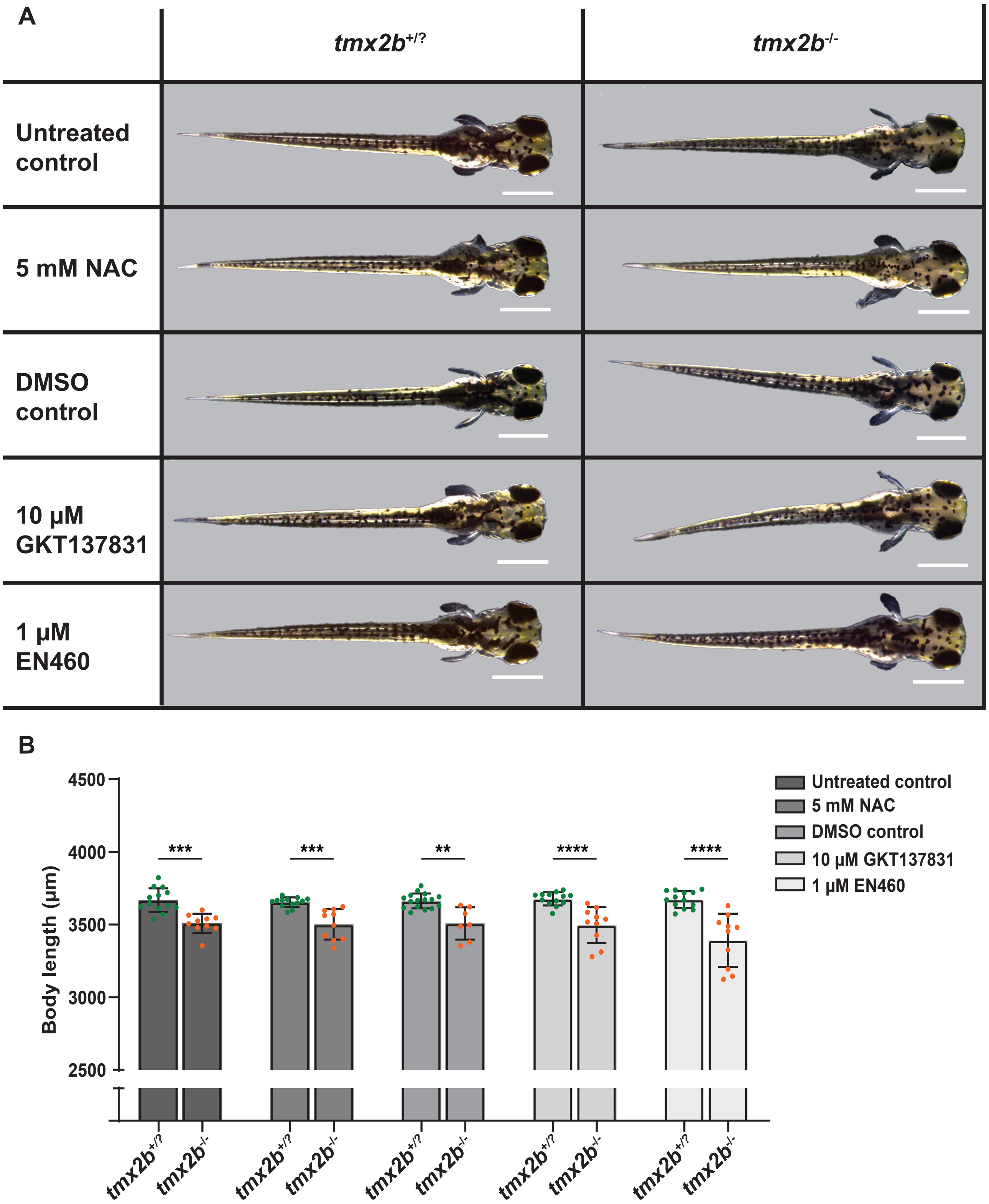

Fig. S13. ROS reducing drug treatments (A) Representative images of 3 dpf tmx2b$^{+/?}$ and tmx2b$^{-/-}$ zebrafish treated with ROS reducing drugs. Scale bars indicate 500 µm. All treated tmx2b$^{-/-}$ zebrafish developed necrosis as is observed by an increased opacity in the brain compared to the tmx2b$^{+/?}$ zebrafish. (B) Body length mea-surements of 3 dpf zebrafish treated with ROS reducing drugs. Untreated, 5 mM NAC, DMSO control, 10 µM GKT137831, 1 µM EN460: tmx2b$^{+/?}$, n=13,13,16,13,13 ; tmx2b$^{-/-}$, n=10, 10, 7, 10, 10 zebrafish. One-way ANOVA with Šídák's multiple comparisons test. Data are represented as mean ± SD. *p < 0.05, **p < 0.01, ***p < 0.001, ****p < 0.0001.

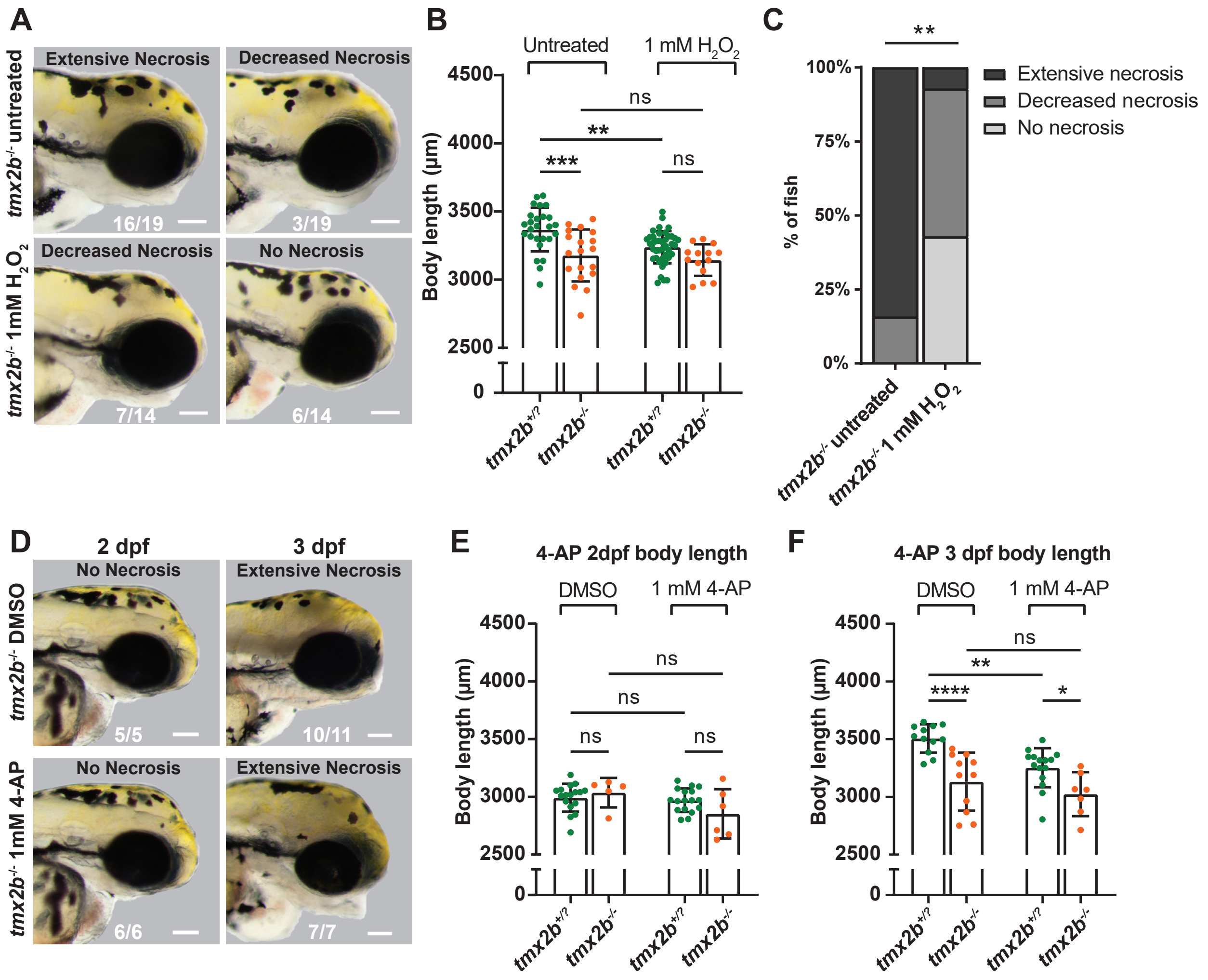

Fig. S14. H_2O_2 and 4-AP treatment. (A) Brightfield images lateral view head of 3 dpf tmx2b$^{-/-}$ untreated (upper images) and treated with 1mM H_2O_2 (lower images). tmx2b$^{-/-}$ fish treated with 1mM H_2O_2 had either decreased necrosis (a reduced opacity compared to the extensive necrosis) or no necrosis at all. Bottom numbers indicate counts of zebrafish with specified genotype and phenotype. Scale bar represents 100 µm. (B) Body length measurements of 3 dpf untreated and 1mM H_2O_2 treated tmx2b$^{+/?}$ and tmx2b$^{-/-}$ zebrafish. Two-way ANOVA, Tukey's multiple comparisons test. (C) Quantification of (A). Fisher's exact test (extensive and decreased necrosis groups were combined for statistical test). (B,C) Untreated, 1mM H2O2 : tmx2b$^{+/?}$, n=26, 41 ; tmx2b$^{-/-}$, n=19, 14 zebrafish. (D) Brightfield images lateral view head of tmx2b$^{-/-}$ zebrafish untreated (upper images) and 1 mM 4-AP treated (lower images) at 2 and 3 dpf. Scale bar represents 100 µm. Bottom numbers indicate counts of zebrafish with specified genotype and phenotype. (E) Body length measurements of 2 dpf DMSO control and 1mM 4-AP treat-ed zebrafish. DMSO control, 1mM 4-AP: tmx2b$^{+/?}$ n=17, 17 tmx2b$^{-/-}$ n=5, 6 zebrafish (F) Body length measurements of 3 dpf DMSO control and 1mM 4-AP treated zebrafish. DMSO control, 1mM 4-AP: tmx2b$^{+/?}$ n=12, 15 tmx2b$^{-/-}$ n=11, 7 zebrafish. Two-way ANOVA, Tukey's multiple comparisons test. Data are represented as mean ± SD. *p < 0.05, **p < 0.01, ***p < 0.001, ****p < 0.0001.

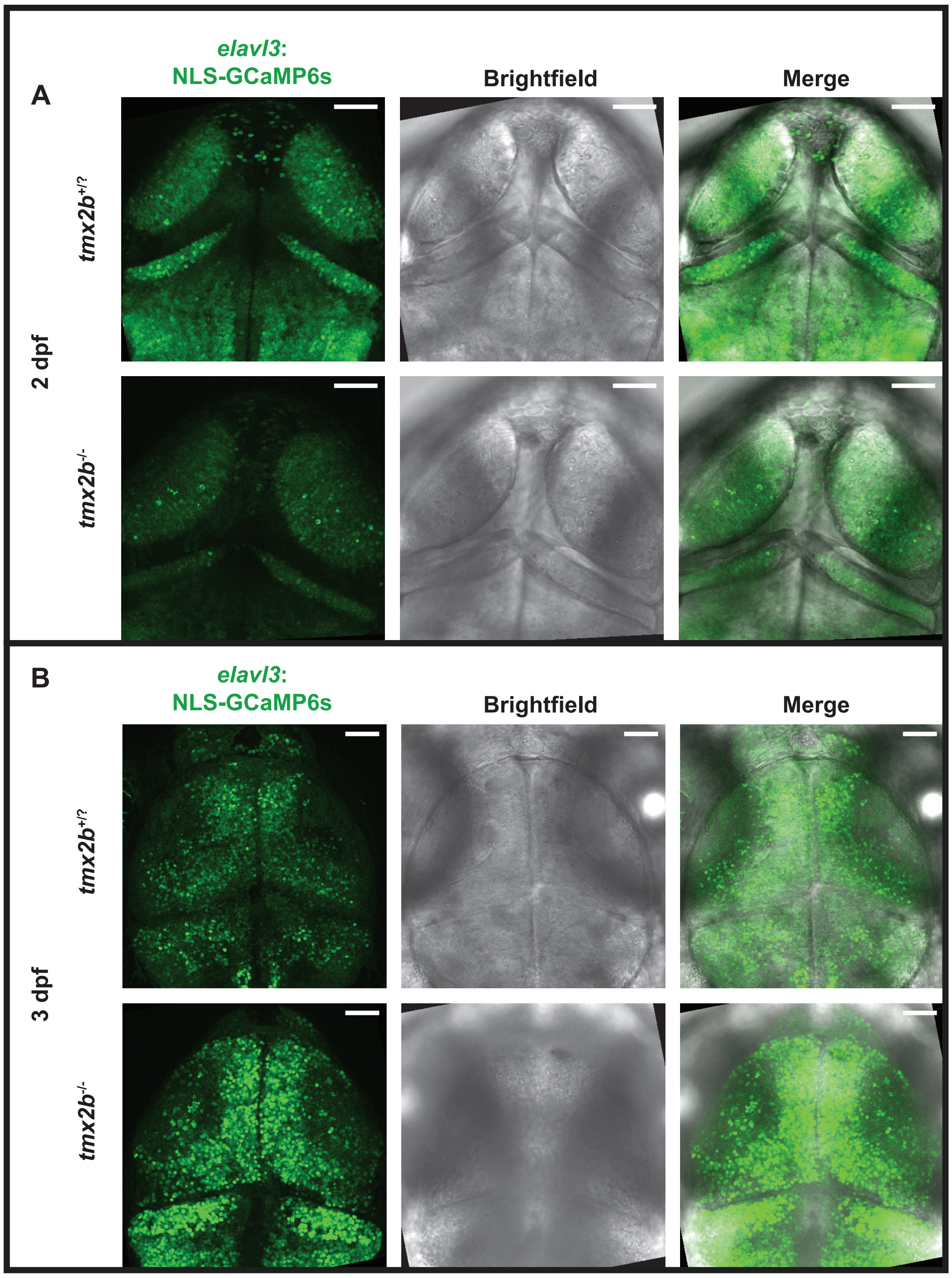

Fig. S15. Calcium imaging in *tmx2b$^{-/-}$* zebrafish brain. (A,B) Representative images of elavl3:NLS-GCaMP6s+ (green) neurons in tmx2b$^{+/?}$ and tmx2b$^{-/-}$ zebrafish at 2 and 3 dpf. 2 dpf: tmx2b$^{+/?}$ n=22, tmx2b$^{-/-}$ n=10 zebrafish. 3 dpf: tmx2b$^{+/?}$ n=16, tmx2b$^{-/-}$ n=16 zebrafish.

Development • Supplementary information

Table S1. Transgenic zebrafish lines

Transgenic zebrafish line	Name in figures and text	Source (PubMed ID)
Tg(ubb:secAnnexinV-mVenus)	*ubb:*secA5-mVenus	20601526
TgBAC(slc17a6b:LOXP-DsRed-LOXP-GFP)	*vglut2:*DsRED	21199937
TgBAC(gad1b:GFP)	*gad1b:*GFP	23946442
Tg(her4.3:EGFP)	*her4.3:*EGFP	17134690
Tg(olig1:NLS-mApple)	*olig1:*NLS-mApple	32066987
Tg(mbp:EGFP-CAAX)	*mbp:*EGFP-CAAX	21880787
Tg(mpeg1:EGFP)	*mpeg1:*EGFP	21084707
Tg(elavl3:NLS-GCaMP6s)	*elavl3:*NLS-GCaMP6s	28740141

Table S2 . Primers Sanger sequencing and allele specific PCR

Target	Fw primer	Rv primer	Allele specific primer	amplicon sizes (bp)
tmx2a WT alelle	CTCCCCTTCTGTTTCATTCACC	GTTTGGAAGTTTGTTGAGGCC	AGGAGTCTCCGTCCTCTCT	500, 230
tmx2a +1 alelle	CTCCCCTTCTGTTTCATTCACC	GTTTGGAAGTTTGTTGAGGCC	GAGTCTCCGTCCTCCGTC	500, 229
tmx2b WT allele	GGCAGTTGTGCTTGTGTTTG	ATTCGCCCTAACCACCTCC	GTGGGCAACATCATCCTCTTC	448, 234
txm2b +7 allele	GGCAGTTGTGCTTGTGTTTG	ATTCGCCCTAACCACCTCC	AACATCATCCTCTTGTGGCCTC	448, 235

Note: For Sanger sequencing only the Fw and Rv primers were used in the PCR reaction

Table S3. Details of drugs used for treatment

Drug	Target	Manufacturer	Catalog number	Concentration (additive)	Start treatment	Refreshing
4-Aminopyridine	Neuronal potassium channel antagonist	Sigma-Aldrich	275875	1 uM (0.1% DMSO)	24 hpf	No
EN460	ERO1 antagonist	MedChem-Express	HY-12837	1 uM (0.1% DMSO)	4 hpf	No
GKT137831	NOX4 antagonist	MedChem-Express	HY-12298	10 uM (0.1% DMSO)	4 hpf	No
N-Acetyl-L-cysteine	General antioxidant	Sigma-Aldrich	A7250	5 mM	4 hpf	No
Hydrogen Peroxide (H_2O_2)	Reactive oxygen species	Sigma-Aldrich	216763	1 mM	6 hpf	2x/day
Ethyl 3-aminobenzoate methanesulfonate salt (Tricaine)	Neuronal voltage-gated sodium channel antagonist	Sigma-Aldrich	A5040	0.008% (m/v)	50 hpf	No

Refreshing indicates refreshing of the medium containing the drug

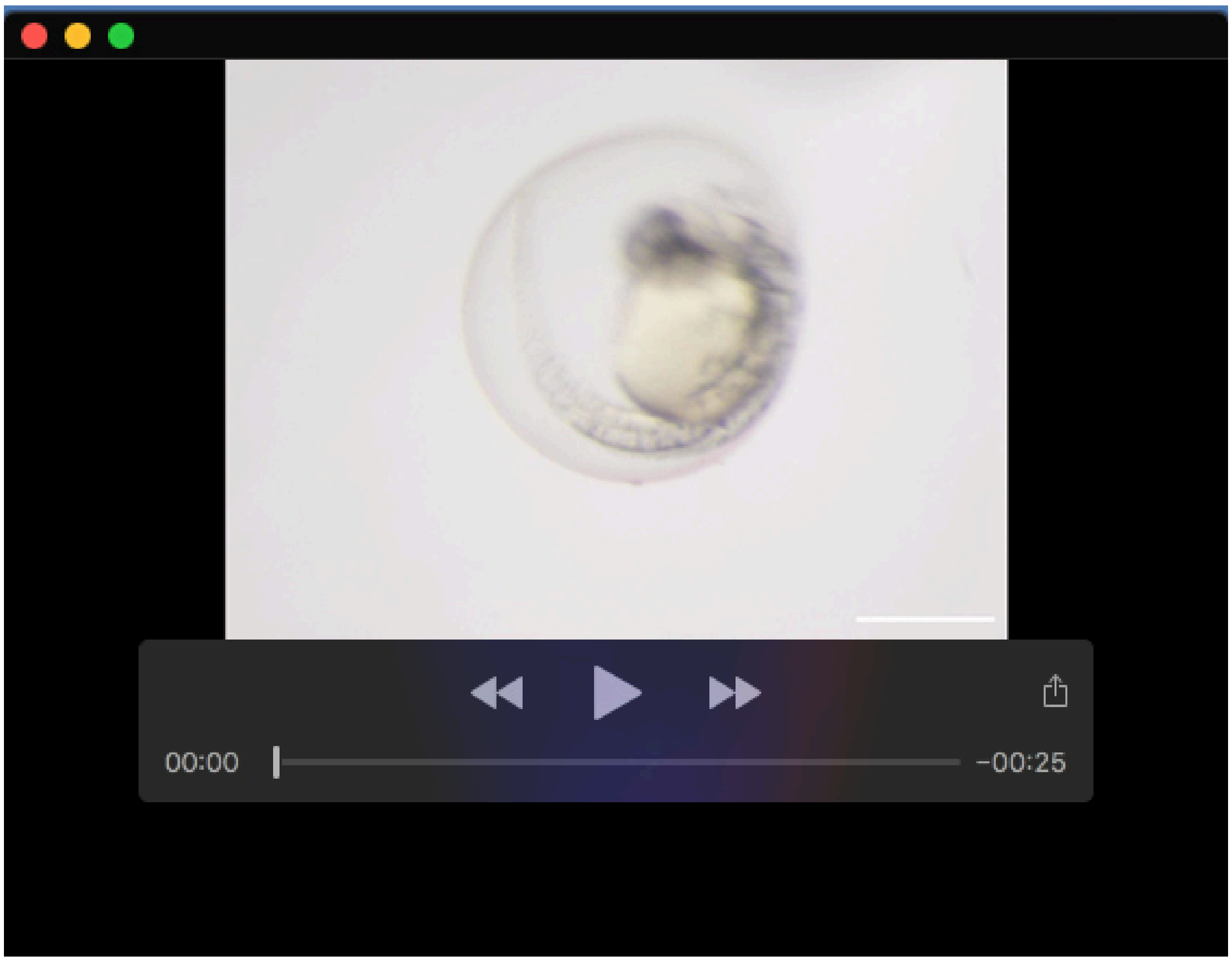

Movie 1. Touch response of *tmx2b*$^{+/?}$ zebrafish at 1 dpf

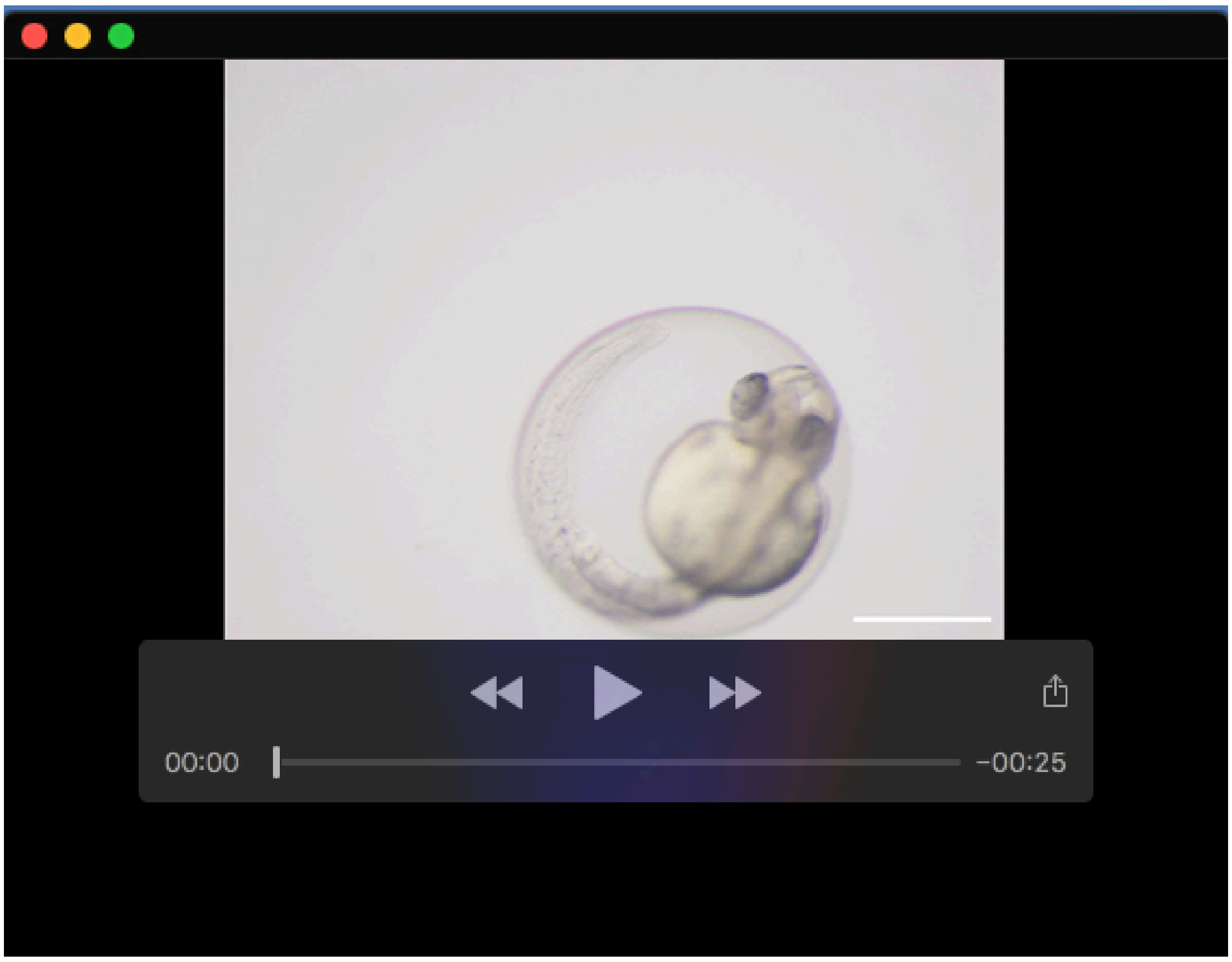

Movie 2. Touch response of *tmx2b*$^{-/-}$ zebrafish at 1 dpf

Development • Supplementary information

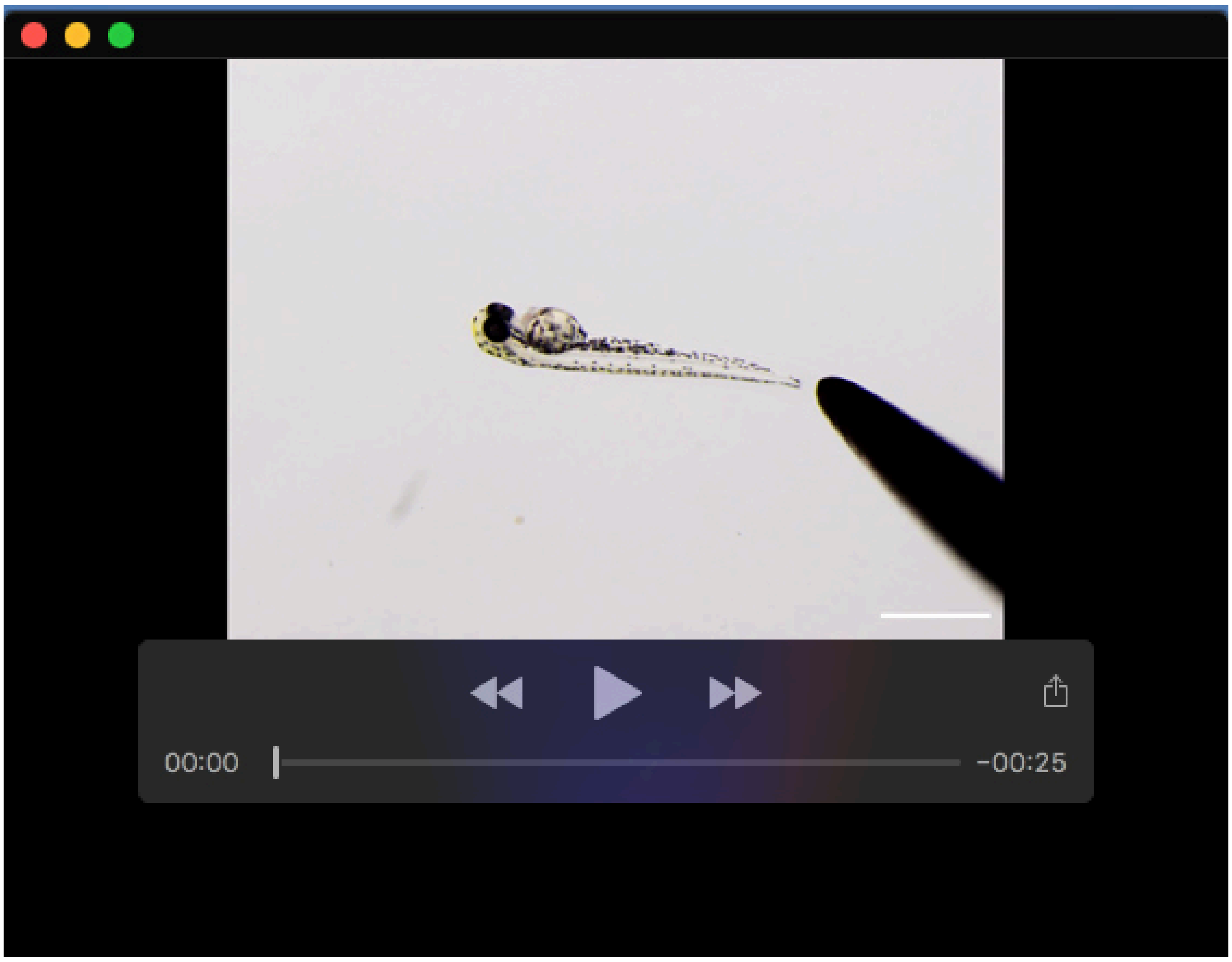

Movie 3. Touch response of *tmx2b*$^{+/?}$ zebrafish at 2 dpf

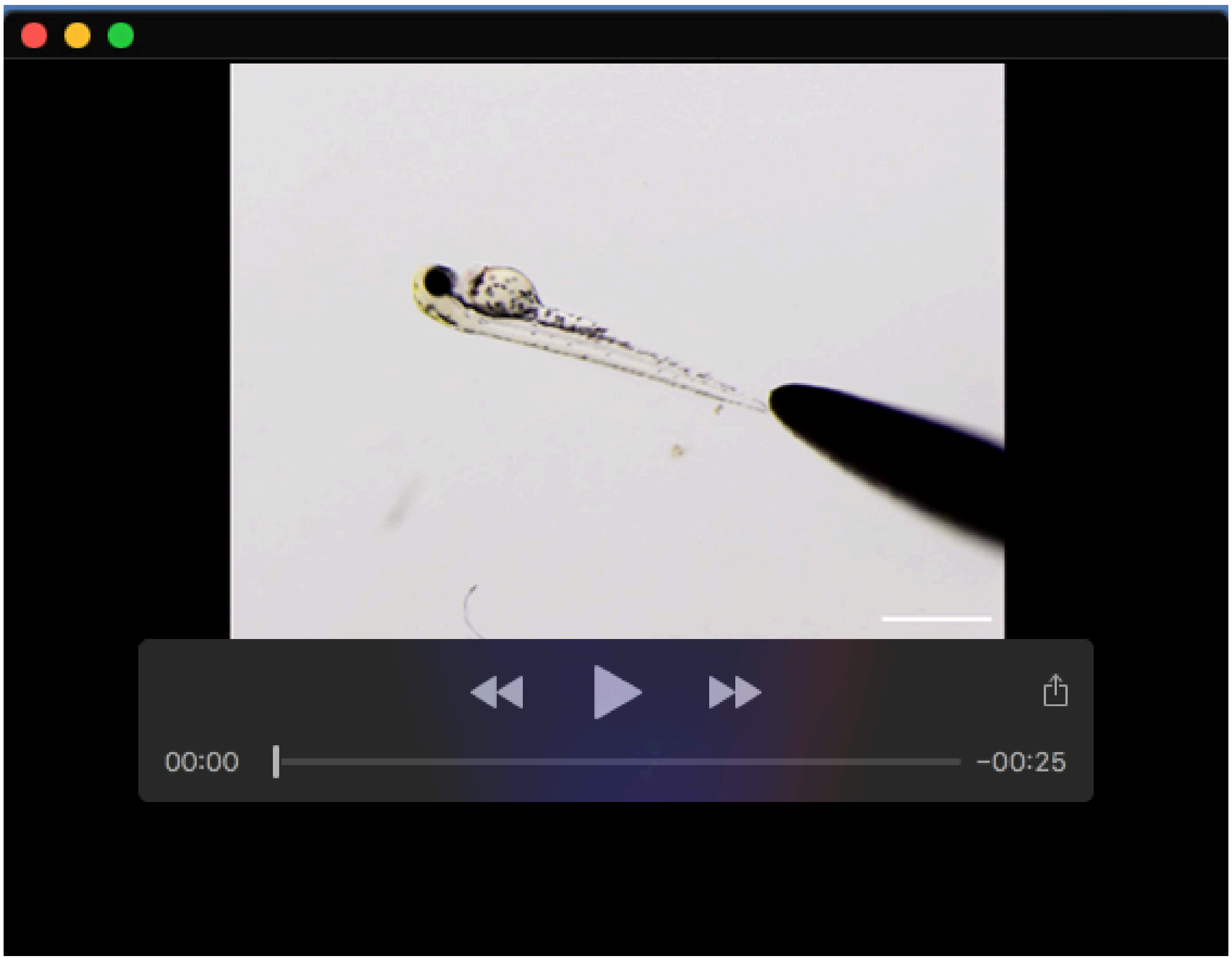

Movie 4. Touch response of *tmx2b*$^{-/-}$ zebrafish at 2 dpf

Development • Supplementary information

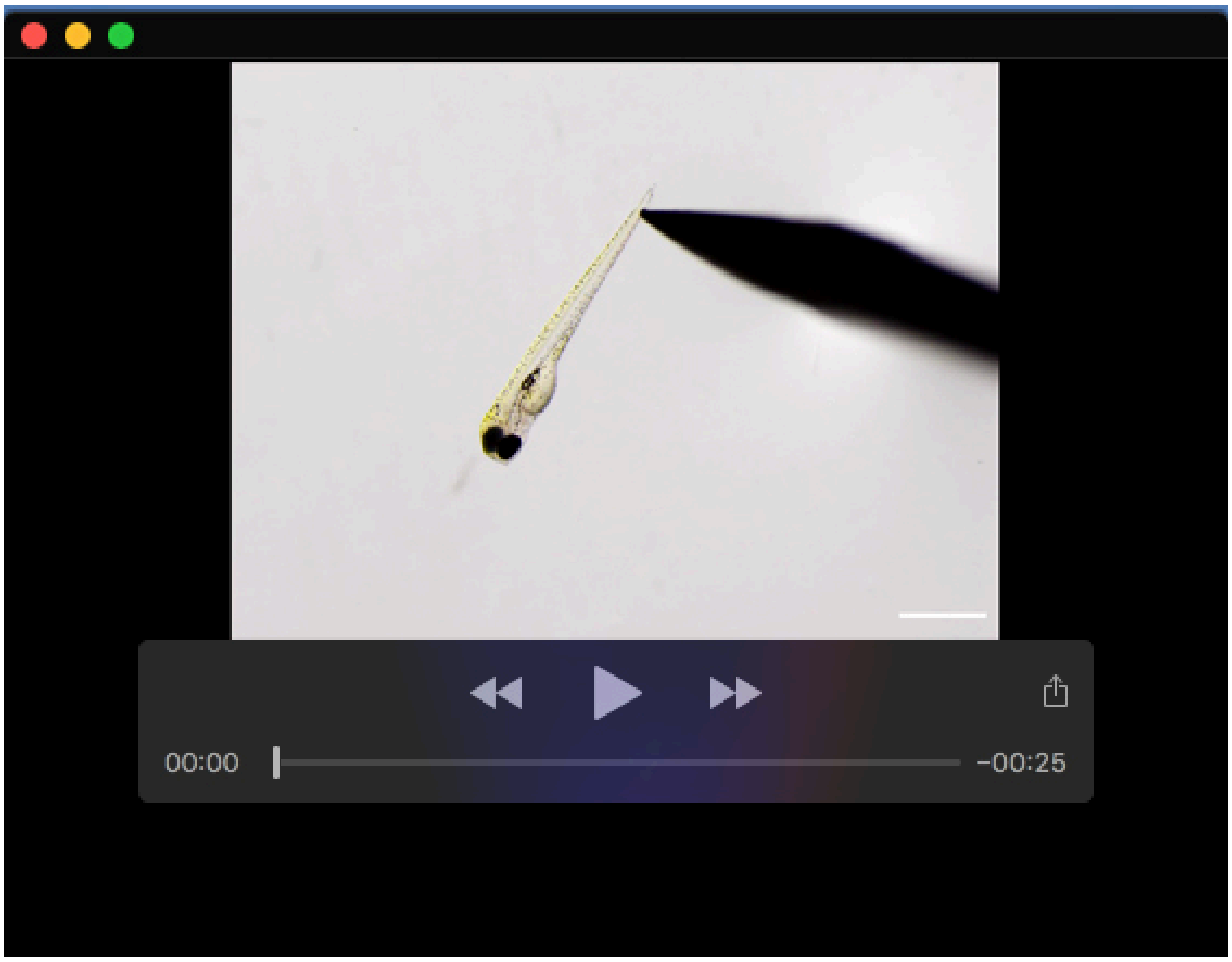

Movie 5. Touch response of *tmx2b*$^{+/?}$ zebrafish at 3 dpf

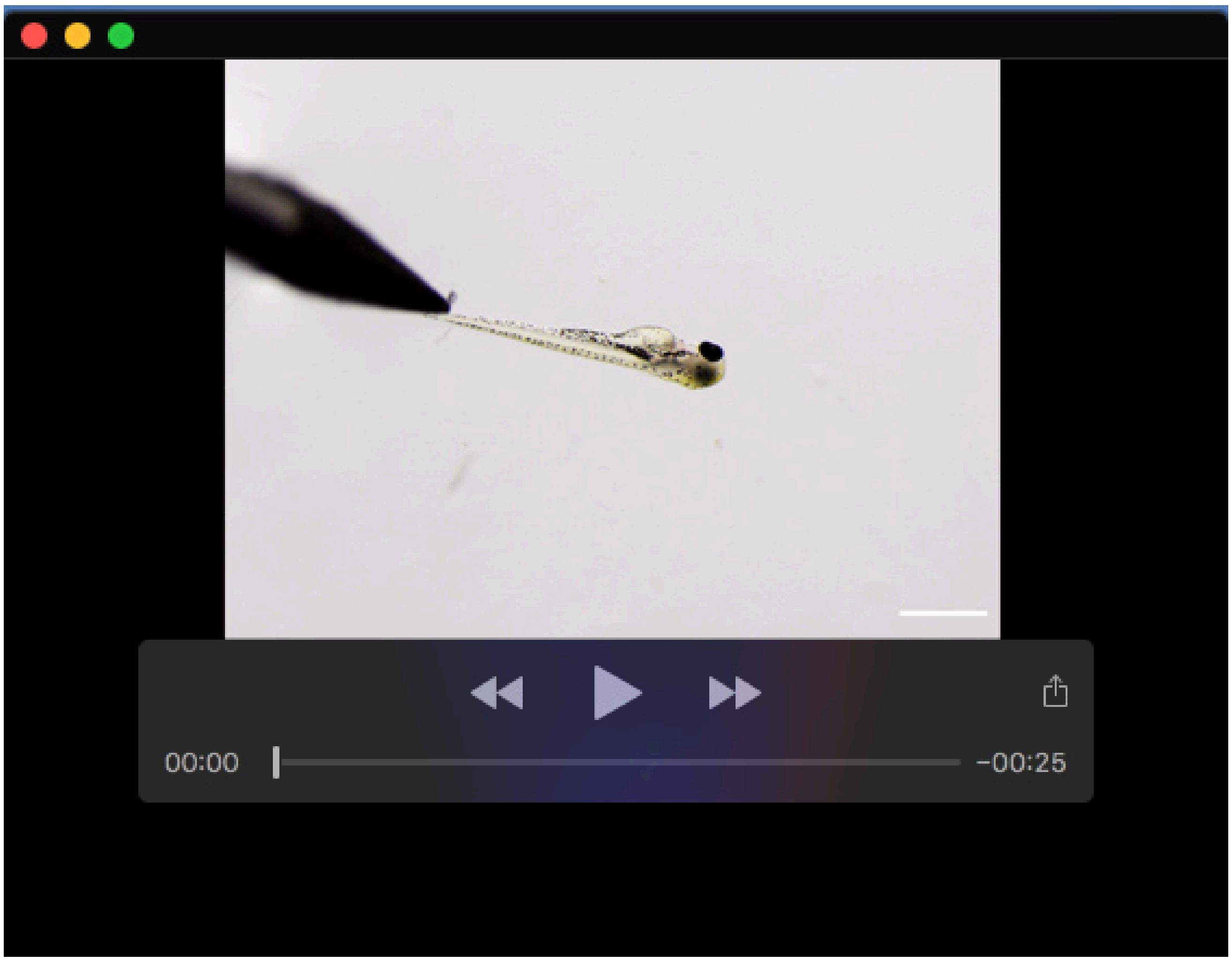

Movie 6. Touch response of *tmx2b*$^{-/-}$ zebrafish at 3 dpf showing no movement upon touch

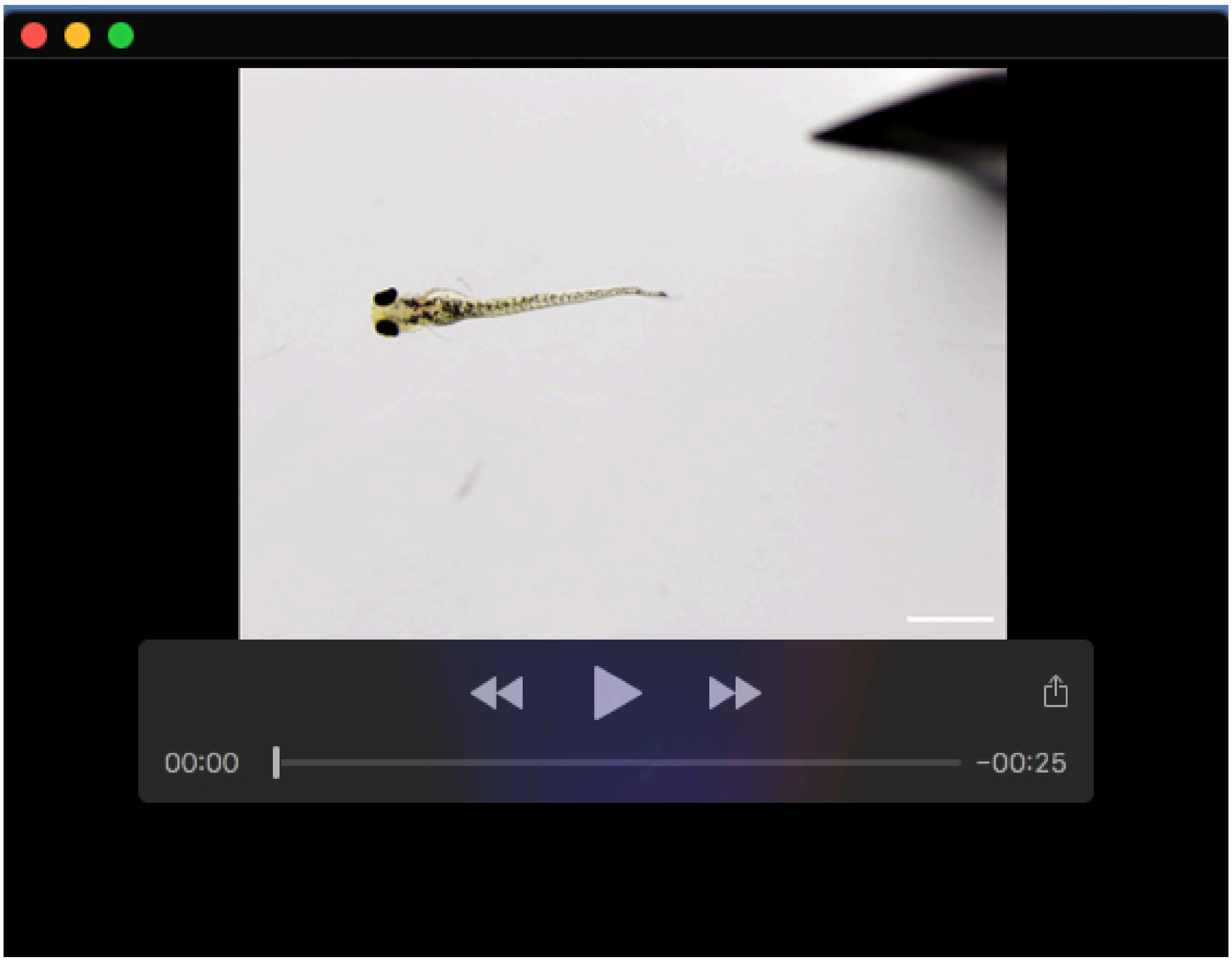

Movie 7. Touch response of *tmx2b*$^{-/-}$ zebrafish at 3 dpf showing a delayed movement upon touch

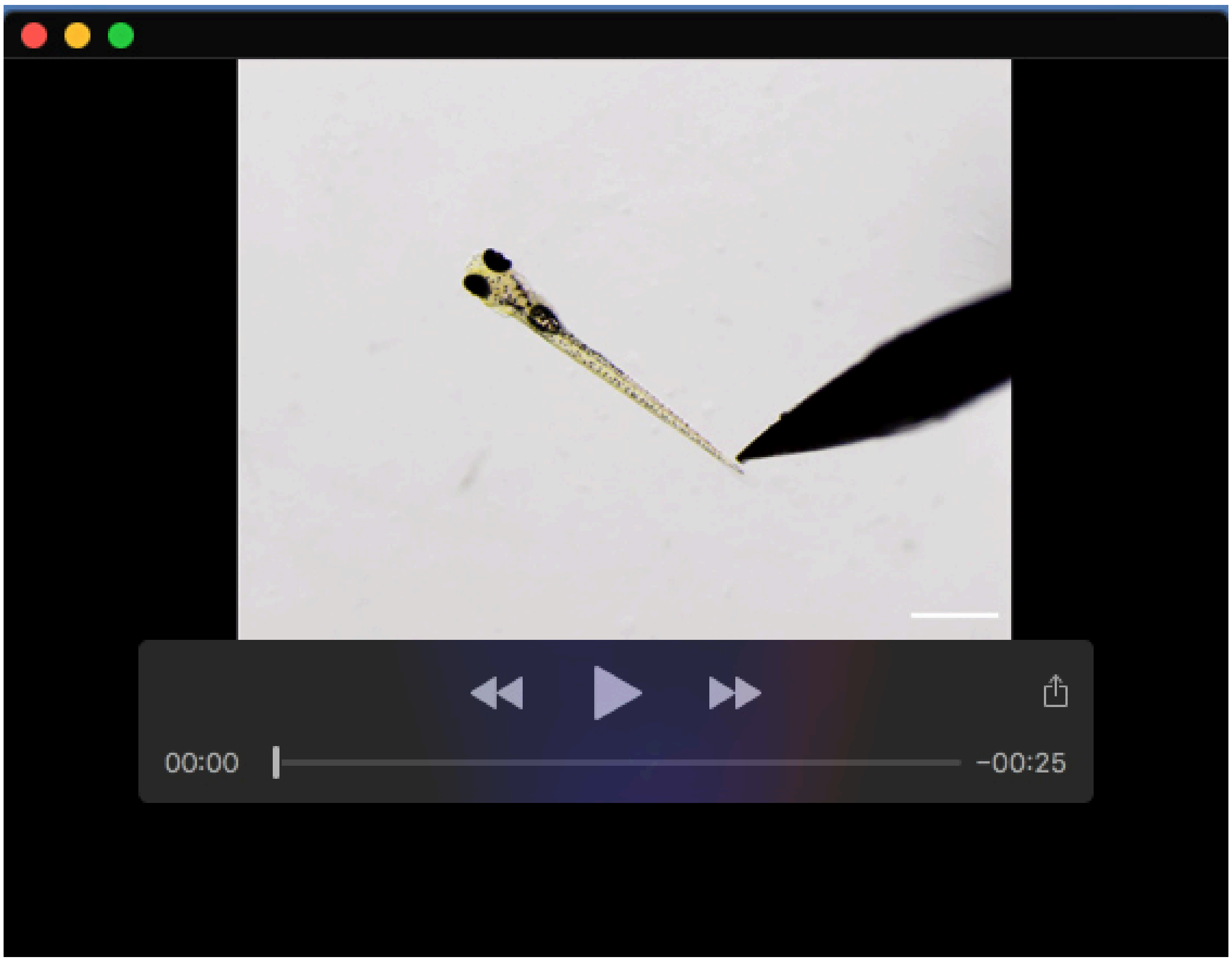

Movie 8. Touch response of *tmx2b*$^{+/?}$ zebrafish at 4 dpf

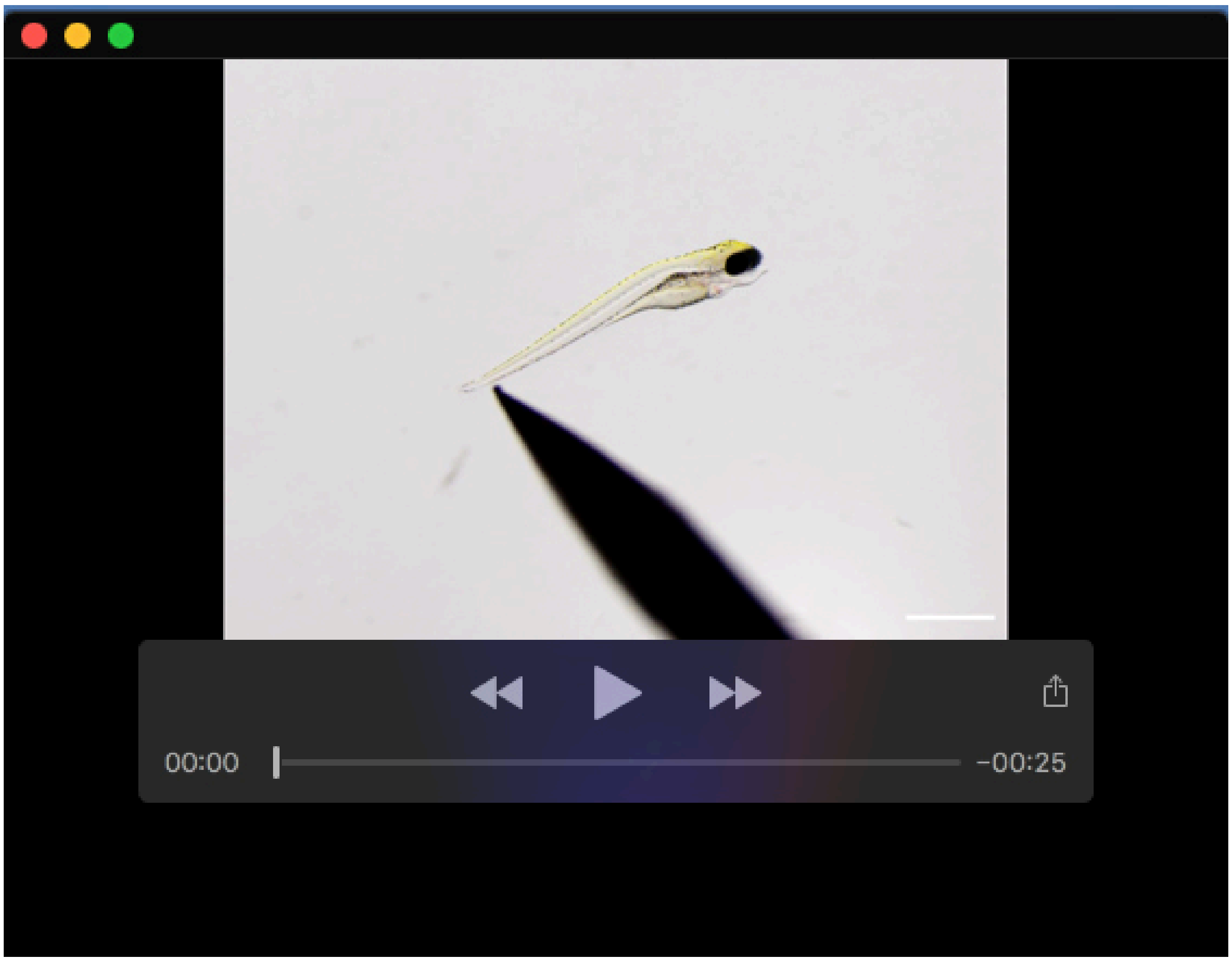

Movie 9. Touch response *tmx2b*$^{-/-}$ zebrafish at 4 dpf

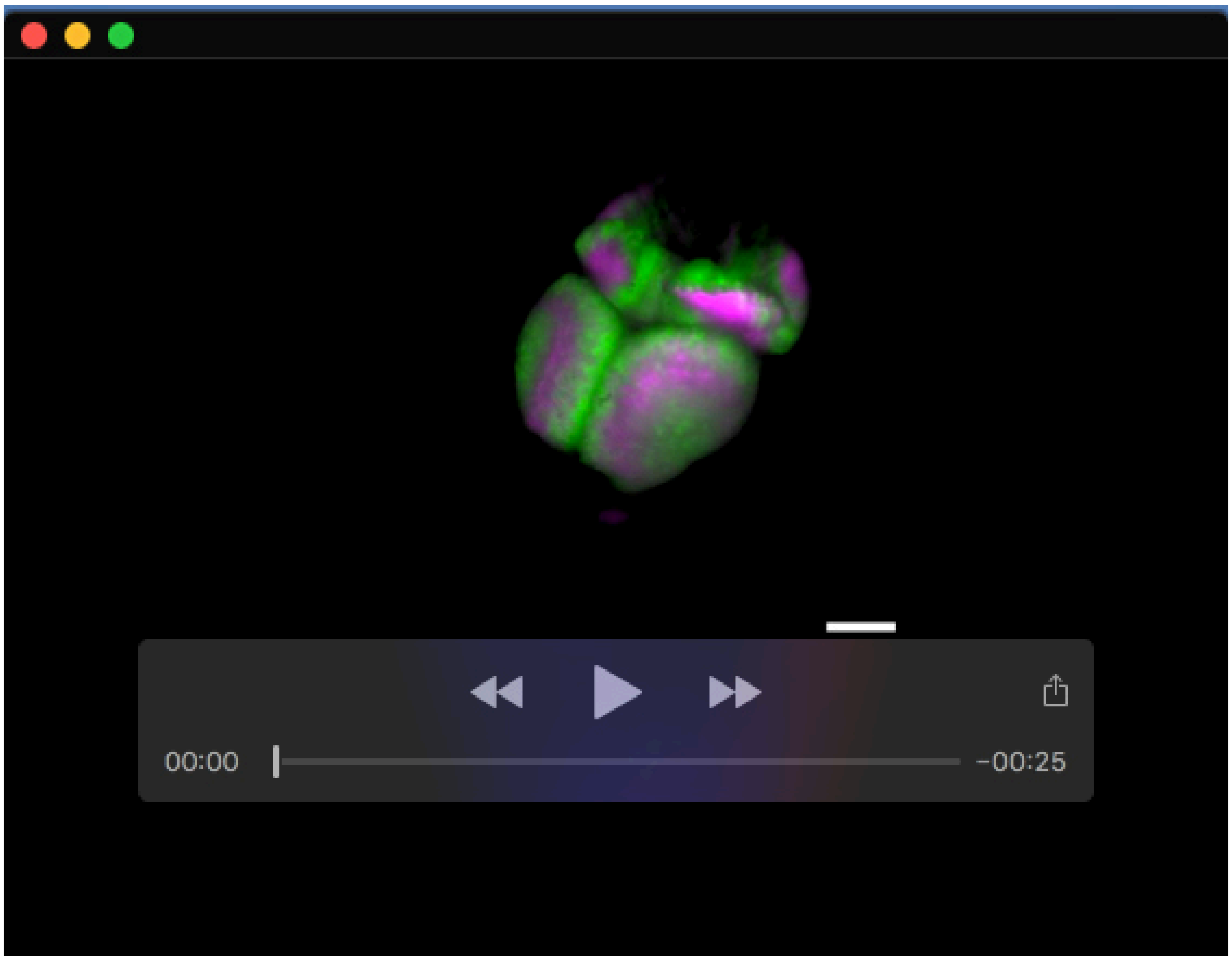

Movie 10. Time-lapse imaging from 58 hpf till 75 hpf of ***tmx2b***$^{+/?}$ zebrafish brain

Development • Supplementary information

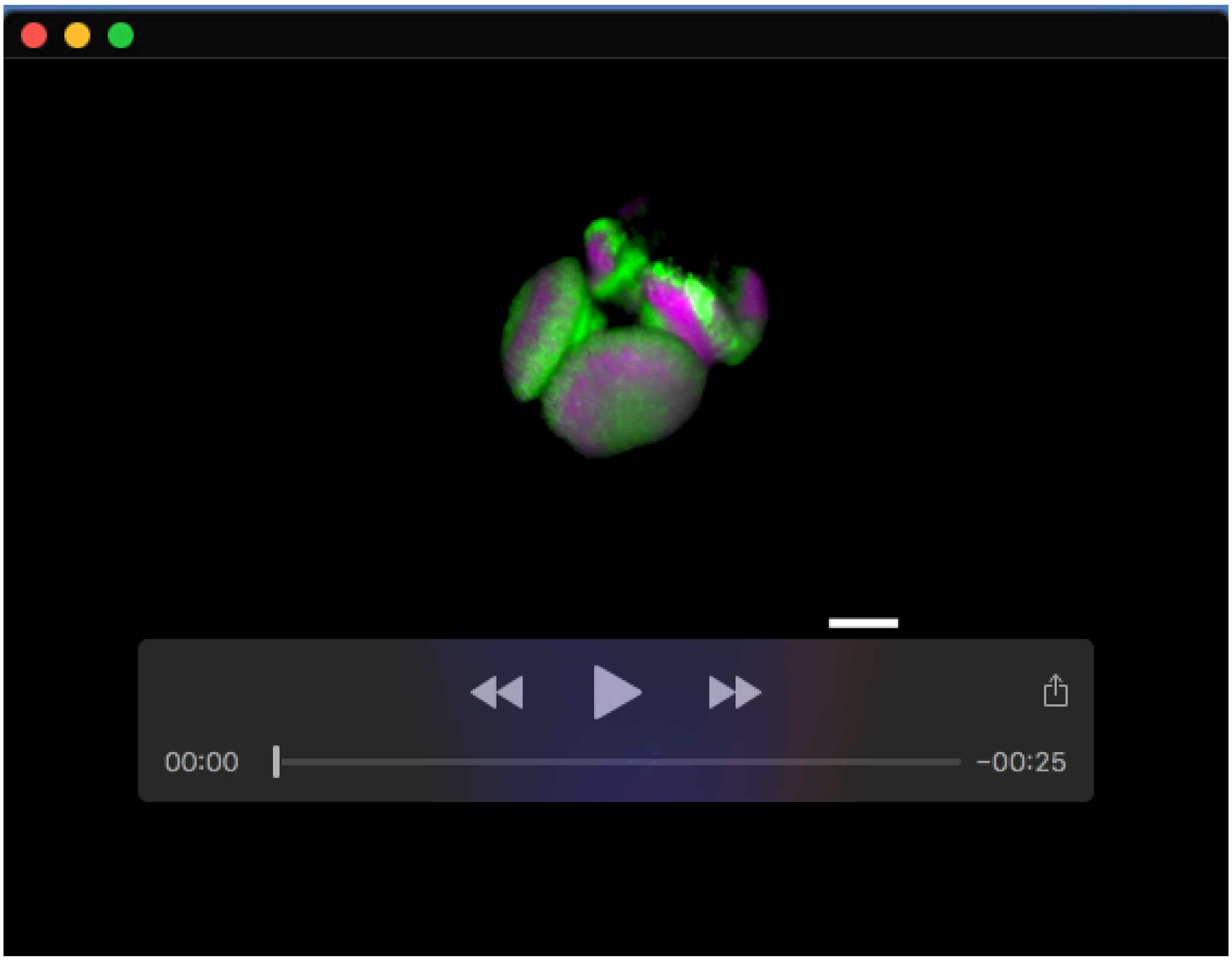

Movie 11. Time-lapse imaging from 58 hpf till 75 hpf of ***tmx2b***$^{-/-}$ zebrafish brain

Development • Supplementary information
